# Lung and cardiac ultrasound for respiratory distress in the elderly: study protocol of the LUC REED stepped-wedge cluster randomised trial

**DOI:** 10.1136/bmjopen-2025-104715

**Published:** 2025-08-16

**Authors:** Frederic Balen, Manon Hebrard, Clément Delmas, Xavier Dubucs, Elise Noel-Savina, Nadège Costa, Jason Shourick, LUC REED investigators, Sandrine Charpentier

**Affiliations:** 1 Emergency Department, CHU Toulouse, Toulouse, France; 2 CERPOP, Toulouse, France; 3 Intensive Cardiac Care Unit, CHU Toulouse Pôle cardiovasculaire et métabolique, Toulouse, France; 4 REICATRA, CHU Toulouse, Toulouse, France; 5 CHU Toulouse, Toulouse, France; 6 Department of Pneumology, CHU Toulouse, Toulouse, France; 7 Healh Economics Unit, CHU Toulouse, Toulouse, France; 8 Department of Epidemiology and Public Health, CHU Toulouse, Toulouse, France

**Keywords:** ULTRASONOGRAPHY, Respiratory Distress Syndrome, Frail Elderly, Emergency Service, Hospital

## Abstract

**Introduction:**

Dyspnea is a common chief complaint leading to emergency department (ED) visits. Multiple conditions may cause or be associated with dyspnoea, including bacterial pneumonia, acute heart failure (AHF), exacerbation of chronic obstructive pulmonary disease (COPD) or asthma and pulmonary embolism. Each of these diagnoses has a specific treatment recommended by international guidelines. Inappropriate treatment in the ED is more frequent among elderly patients and is independently associated with in-hospital mortality. Point-of-care ultrasound is immediately available at the bedside. Lung and cardiac ultrasound (LuCUS) offers excellent diagnostic accuracy for bacterial pneumonia, AHF and COPD exacerbations, even in elderly patients. The primary objective of the LUC REED trial is to evaluate the impact of a LuCUS-guided strategy versus standard care on reducing inappropriate treatment of dyspnoea in elderly ED patients.

**Methods and analysis:**

The LUC REED trial is a prospective, interventional, multicentre, stepped-wedge randomised controlled trial designed to assess the superiority of a LuCUS-guided strategy over standard care in ensuring treatment appropriateness for dyspnoea in elderly ED patients. The study will include 504 patients over 2 years. Patients aged >65 years presenting with acute dyspnoea and signs of severity (respiratory rate ≥22 and SpO_2_ <92% on room air) will be enrolled. Each ED (cluster) will start with a control phase. Every 3 months, one centre will transition to the intervention phase (LuCUS-guided strategy). The primary outcome is treatment inappropriateness within the first hour after inclusion, assessed by comparing administered treatment to the final diagnosis adjudicated by two experts.

**Ethics and dissemination:**

Ethics final approval was obtained from the Institutional Review Board of France—Est IV on 4 April 2025 (2024-A01678-39). Results will be published in peer-reviewed international journals.

**Trial registration number:**

NCT06807983.

**Strengths and limitations of this study**

- The LUC REED trial is a prospective, interventional, multicentre, stepped-wedge randomised.
- The stepped-wedge randomisation is probably an easier way to randomise patients in emergency department settings.
- The stepped-wedge design offers the advantage of allowing each centre to serve as its control, which helps account for variability between centres.

## Introduction

 Dyspnoea is a common reason for emergency department (ED) visits, accounting for more than 5% of admissions outside the winter viral season.^1^ Multiple conditions can cause or be associated with dyspnoea, including bacterial pneumonia, acute heart failure (AHF), exacerbations of chronic obstructive pulmonary disease (COPD) or asthma and pulmonary embolism.^1^,^4^ Each of these diagnoses has a specific treatment recommended by international guidelines. In the ED, bacterial pneumonia is treated with antibiotics,^5^ AHF with furosemide and nitrates,^6^ COPD exacerbations with β2-agonists,^7^ asthma with β2-agonists and corticosteroids^8^ and pulmonary embolism with therapeutic anticoagulation.^9^

In practice, the main challenge for emergency physicians (EPs) is accurately identifying the underlying cause of dyspnoea to initiate appropriate treatment. Clinical signs can be misleading, and the diagnostic workup is often complex.^10^,^12^ However, early treatment is crucial to improving patient outcomes,^13 14^ which can result in a high rate of inappropriate treatments, especially in elderly patients.^4 15 16^ Inappropriate treatment in the ED has been independently associated with in-hospital mortality.^4 15^ While biomarkers can aid in the diagnostic process,^17 18^ they are not immediately available.

In contrast, point-of-care ultrasound is readily available at the bedside. Lung ultrasound (LUS) has excellent diagnostic accuracy for bacterial pneumonia, AHF and COPD exacerbations.^19 20^ When combined with cardiac ultrasound (CUS), its accuracy improves further.^21 22^ Lung and cardiac ultrasound (LuCUS) is particularly promising in more complex populations, such as elderly patients.^23 24^ Its impact on diagnostic accuracy and treatment appropriateness has been evaluated in unselected ED patients with dyspnoea,^2125^,^27^ but its implementation in standard practice remains limited, and the overall level of supporting evidence is moderate. LuCUS may have a greater impact on elderly patients, who are more likely to receive inappropriate treatment for dyspnoea.^4^ Improved diagnosis and treatment could also enhance patient pathways, including outcomes like mortality, days alive outside the hospital and healthcare costs.

The primary objective of the LUC REED trial is to assess the impact of a LuCUS-guided strategy compared with standard care in reducing inappropriate treatment of dyspnoea in elderly ED patients. Secondary objectives include evaluating the impact of LuCUS on diagnostic accuracy, ED and hospital length of stay (LOS), number of days alive outside the hospital at 30 days, 30-day mortality and cost-effectiveness and cost-utility.

## Methods

### Trial design

The LUC REED trial (NCT06807983) is a prospective, interventional, multicentre, stepped-wedge randomised controlled trial designed to assess the superiority of a LuCUS-guided strategy over standard care in ensuring appropriate early treatment of dyspnoea in elderly patients in the ED. The final version of the protocol is available in online supplemental file 1). This protocol follows the Standard Protocol Items: Recommendations for Interventional Trials guidelines.

### Patients and public involvement

Patients and the public were not involved in the design of the research protocol.

### Study settings

Patients will be recruited from seven academic, urban EDs across France. The recruitment period is expected to last 2 years, starting on 3 November 2025, and ending on 2 November 2027. Each ED (cluster) will start with a control phase (standard care). Every 3 months, one ED will be randomly selected to transition to the intervention phase (LuCUS-guided strategy). The trial will therefore proceed in eight 3-month steps, with the final step involving all centres operating under the intervention phase (see online supplemental file 2). The recruitment will conclude after 2 years. A cluster randomised design was chosen because the implementation of the LuCUS-guided strategy in real-life settings is part of the intervention under evaluation. Individual randomisation was therefore not feasible. The stepped-wedge design also offers the advantage of allowing each centre to serve as its control, which helps account for variability between centres. Additionally, this design allows time for retraining and reimplementation of the LuCUS strategy at each transition point.

### Selection of participants

Eligible patients will be aged over 65 years, affiliated with the French social security system, and presenting to the ED with acute dyspnoea (onset <14 days) accompanied by severe signs before or at triage (respiratory rate ≥22 and SpO₂ <92% on room air). The enrolling EP must be the patient’s treating physician and must be trained in LuCUS. Written informed consent from the patient or their legal representative is required for inclusion (online supplemental file 3). In emergencies where this is not possible, patients may be enrolled under emergency inclusion procedures, and consent will be obtained as soon as feasible in accordance with Article L1122-1-2 of the French Public Health Code. Exclusion criteria include: dyspnoea secondary to thoracic trauma, dyspnoea related to COVID-19, known pulmonary fibrosis or lung cancer, prior administration of specific treatment for dyspnoea before inclusion, immediate need for endotracheal intubation, patients identified as being at end-of-life and individuals under legal guardianship or deprived of liberty.

### Intervention

During the control period, diagnostic workup and treatment will be performed at the discretion of the treating EP. Information regarding the treatments administered and the suspected diagnoses will be collected 1 hour after inclusion, as well as after the patient’s ED stay, in both the control and intervention periods.

**Figure 1:**
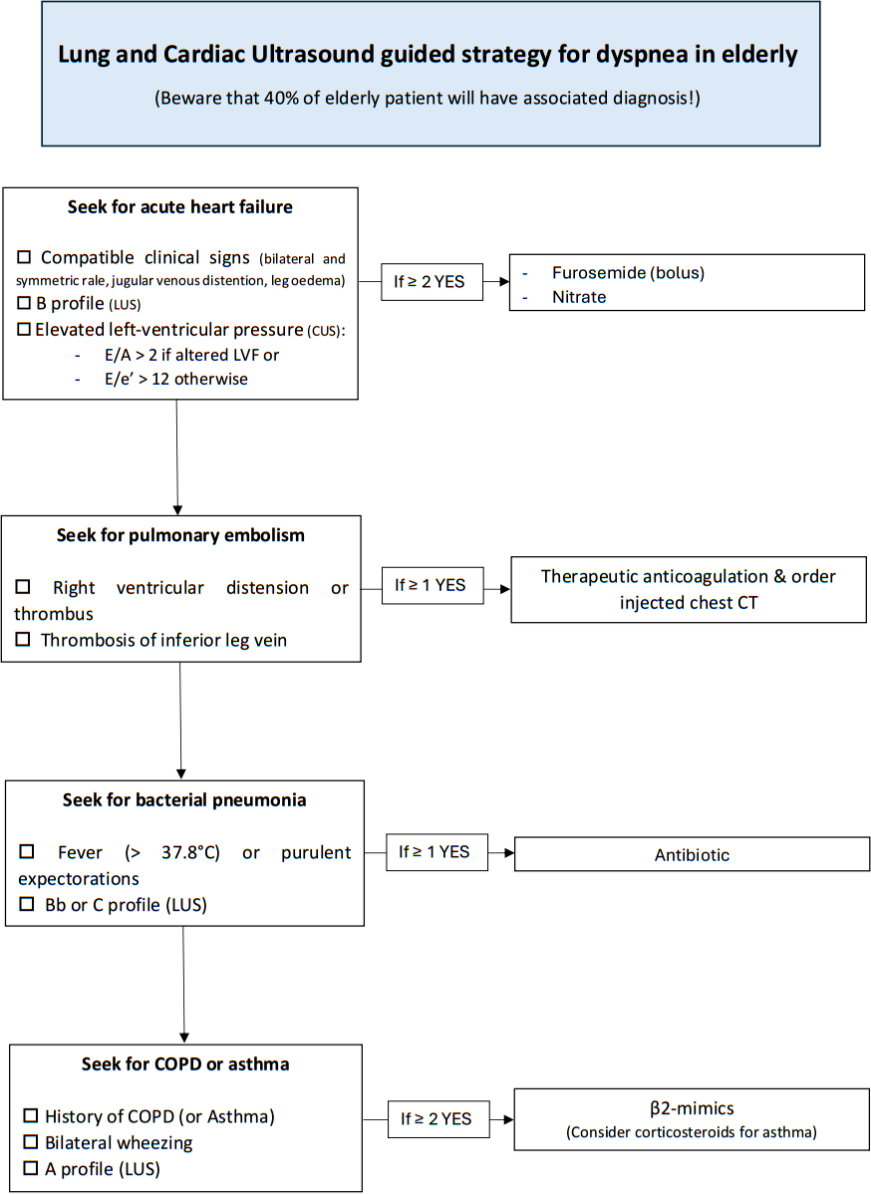
LuCUS guided the strategy in the intervention group. COPD, chronic obstructive pulmonary disease; CUS, cardiac ultrasound; LUS, lung ultrasound; LVF, left ventricule function.

When an ED transitions to the intervention phase, LuCUS semiology will be reviewed with the medical team. The LuCUS-guided strategy will then be implemented (see figure 1), following the criteria below:

- AHF is likely and should be treated using furosemide and nitrate^6^ if two of the following criteria are found:
  - Compatible clinical signs (bilateral and symmetric rale, jugular venous distention, leg oedema).^10^
  - B profile at LUS.^19 20 28^
  - Elevated left-ventricular pressure at CUS (E/A >2 at mitral flow assessment if Left Ventricule Function (LVF) altered or E/e’ >12 otherwise).^21 22^
- Pulmonary embolism is likely and should be treated using therapeutic anticoagulation^9^ if one of the following signs is found:
  - Previously unknown right ventricular distension or thrombus.^29^
  - Deep vein thrombosis in the lower limbs.^28^
- Bacterial pneumonia is likely and should be treated using antibiotics^5^ if one of the following signs is found:
  - Fever (>37.8°C) or purulent sputum.^12^
  - Bb or C profile at LUS.^19 20 28^
- COPD or asthma exacerbation is likely and should be treated using β2-mimics^7 8^ if two of the following criteria are found:
  - History of documented COPD or asthma.
  - Bilateral wheezing.
  - A profile at LUS.^19 20 28^

This standardised LuCUS-based protocol will guide treatment decisions during the intervention phase to improve diagnostic accuracy and reduce inappropriate therapy.

### Outcomes

The primary outcome is the appropriateness of the treatment for dyspnoea administered within the first hour after inclusion. Treatment will be evaluated as appropriate or inappropriate by comparing the actual treatment given to the final diagnosis. This final diagnosis will be adjudicated by two independent experts—a pulmonologist (EN-S) and a cardiologist (CD)—based on a standardised report, which includes clinical presentation, laboratory results and imaging (eg, chest X-ray or other available chest imaging). These experts will be blinded to the LuCUS findings. In the case of disagreement between the two experts, a third adjudicator (an EP, XD) will determine the final diagnosis using the same dataset.

Once the final diagnosis is established, the treatment administered in the first hour will be evaluated against current international guidelines (see table 1). Treatment is defined as appropriate if it aligns with the recommendations. Appropriate treatment of AHF includes furosemide and nitrate.^6^ Appropriate treatment of bacterial pneumonia is based on antibiotics.^5^ Appropriate treatment of COPD exacerbation is β2-mimetics, antibiotics or corticosteroids, which are possible (ie, they do not classify patients as having an inappropriate treatment if used in this indication).^7^ Appropriate treatment of asthma is β2-mimetics and corticosteroids.^8^ Appropriate treatment of pulmonary embolism is therapeutic anticoagulation.^9^ If a patient presents with two concurrent diagnoses (eg, AHF and pneumonia), a combination of both treatments (eg, furosemide and antibiotics) is considered appropriate. Failure to administer any recommended treatment is considered inappropriate (under-treatment). Administration of a treatment not recommended for the final diagnosis (eg, β2-agonists for AHF) is also classified as inappropriate (over-treatment). This classification has been used in a prior study that showed an increase in-hospital mortality in the ‘inappropriate treatment’ group.^4^

**Table 1:** Appropriate and inappropriate treatments

Final diagnosis	Treatments initiated during the first hour				
	Furosemide+N	β2-mimetics	Antibiotics	Corticosteroids	Anticoagulation
Acute heart failure	Appropriate	Inappropriate	Inappropriate	Inappropriate	Possible
Bacterial pneumonia	Inappropriate	Inappropriate	Appropriate	Inappropriate	Inappropriate
COPD exacerbation	Inappropriate	Appropriate	Possible	Possible	Inappropriate
Asthma	Inappropriate	Appropriate	Inappropriate	Appropriate	Inappropriate
Pulmonary embolism	Inappropriate	Inappropriate	Inappropriate	Inappropriate	Appropriate
Others	Inappropriate	Inappropriate	Possible*	Inappropriate	Inappropriate

* if ‘Other’ is an infection: appropriate.

COPD, chronic obstructive pulmonary disease; N, nitrate.

The secondary end-points are diagnosis accuracy (compared with final diagnosis), ED and hospital LOS, number of days alive outside the hospital at 30 days, 30-day mortality and direct and indirect medical costs, looking at French social security spending for the patient.

### Statistical analysis

All statistical analyses will be conducted on an intention-to-treat basis. Quantitative variables will be described using counts, means, SD and percentile distributions. Because the study uses a cluster-randomised design, randomisation may not ensure equal distribution of potential confounding variables between the two strategies. Therefore, baseline characteristics will be compared between the intervention and control groups. The intra-cluster correlation coefficient (ICC) will be calculated. If the ICC exceeds 0.062, a post hoc power analysis will be performed. For analysis of the primary outcome, a mixed-effects logistic regression model will be used to compare treatment inappropriateness between the intervention and control strategies. The model will include centre (ED) as a random intercept and will be adjusted for the time of recruitment and potential confounders, such as age, sex and relevant medical history. This same approach will be applied to the analysis of secondary outcomes. Missing data will be handled using multiple imputation techniques. A p value <0.05 will be considered statistically significant.

Patient care costs will be described in both arms. Quantitative cost variables will be summarised using means, SD, minimum and maximum values, quartiles and medians. Comparisons between groups will use appropriate statistical tests (Student’s t-test or Mann–Whitney U test, as applicable). To examine socioeconomic disparities, data on patient income level, education and pre-retirement occupational category will be collected. Mixed-effects models will be used to assess the influence of these variables on healthcare costs. A net equity impact analysis will be conducted to estimate how these characteristics affect cost-effectiveness. Both deterministic and probabilistic sensitivity analyses will be conducted. An acceptability curve will be created to assess the probability that the intervention is cost-effective at various willingness-to-pay thresholds set by the payer. Budget impact will be estimated using a closed-cohort approach and modelled via a decision tree framework.

### Sample size

Inappropriate treatment of dyspnoea in this population is estimated to occur at a 35% rate.^4 15^ We estimate the intervention can reduce it to 17.5%.^21 27^ The number of steps (n=8), the number of clusters (n=7), the number of cluster randomised by step (n=1), the intra-cluster correlation (ICC=0.01), a power of 80% and an α=5% were considered for sample size calculation using Hemming and Taljaard estimation method for stepped-wedge trials.^30^ Considering that 10% of patients may be lost, we calculate the sample size to be 504 patients.

## Ethics and dissemination

Ethics final approval was obtained from the Institutional Review Board of France—Est IV on 4 April 2025 (2024-A01678-39). The study will start in 2025. The National Commission on Informatics and Liberty (France) gave its approval on 4 December 2024 (Re#924228). The results of this study will be presented at national meetings and published in international peer-reviewed journals. The principal publication from the study will be in the name of the LUC REED investigators with full credit assigned to all active, collaborating investigators, research coordinators and institutions. The results of the medico-economic analysis will be published in a second article.

## Discussion

LuCUS already appears to be a relevant tool in dyspneic patients in the ED.^25^,^27^ However, it seems that LuCUS remains underused in standard practice.^4^ Improving the level of evidence in favour of its use may help to increase its practice. Indeed, LuCUS performances for the diagnosis of the origin of dyspnoea are already established.^20^ However, there are still a few studies that show an improvement in patients’ outcomes. Reducing inappropriate treatment appears to be an interesting first step to study the impact of LuCUS on morbimortality of dyspneic patients in the ED. We choose to focus our study on the population that is most exposed to inappropriate treatment (ie, elderly patients). Indeed, improving diagnosis workup in patients who did not present a diagnosis challenge would be futile. Showing an improvement in the pathway of elderly patients attending ED for dyspnoea may lead to an upgrade in class recommendations of LuCUS use.

## Supplementary material

10.1136/bmjopen-2025-104715 online supplemental file 1

10.1136/bmjopen-2025-104715 online supplemental file 2

10.1136/bmjopen-2025-104715 online supplemental file 3
