# Correction: Lung and cardiac ultrasound for respiratory distress in the elderly: study protocol of the LUC REED stepped-wedge cluster randomised trial

**DOI:** 10.1136/bmjopen-2025-104715corr1

**Published:** 2025-10-28

 Balen F, Hebrard M, Delmas C, *et al*. Lung and cardiac ultrasound for respiratory distress in the elderly: study protocol of the LUC REED stepped-wedge cluster randomised trial. *BMJ Open* 2025;15:e104715. doi:10.1136/bmjopen-2025–104715

This article was previously published with an error. Below minor discrepancies between the French version of the LUC REED protocol and this version have been identified.

- ‘Respiratory Rate ≥22 or SpO₂ <92% on room air’ has been corrected to ‘Respiratory Rate ≥22 and SpO₂ <92% on room air’ in the Methods and analysis section of the Abstract and in the Selection of participants section under Methods.
- ‘… (onset <7 days)’ has been corrected to ‘… (onset <14 days)’ in the Selection of participants section under Methods.
- ‘… starting on 8 September 2025, and ending on 7 September 2027’ has been updated to ‘starting on 3 November 2025, and ending on 2 November 2027’ in the Study settings section under Methods.

