# Supplementary material for: Lung and cardiac ultrasound for respiratory distress in the elderly: study protocol of the LUC REED stepped-wedge cluster randomised trial
Source: BMJ Open. 2025 Aug 16;15(8):e104715. doi: 10.1136/bmjopen-2025-104715 (PMC12359426; doi:10.1136/bmjopen-2025-104715)
Supplement: online supplemental file 1 [file bmjopen-15-8-s001.pdf]

Impact d'une stratégie de prise en charge de la détresse respiratoire aiguë chez le sujet âgé basée sur l'utilisation de l'échographie cardiopulmonaire

LUC REED

RC31/23/0386

PROTOCOLE DE RECHERCHE INTERVENTIONNELLE IMPLIQUANT LA PERSONNE HUMAINE *(catégorie 2)*

Version n°1.4 du 10/03/2025

Numéro ID-RCB: 2024-A01678-39

Cette recherche interventionnelle a obtenu le financement du Ministère de la Santé (Programme Hospitalier de Recherche Clinique Interrégional 2023)

Promoteur :
CHU Toulouse, Hôtel-Dieu, 2 rue Viguerie, TSA 80035, 31059 Toulouse cedex

Investigateur coordonnateur :
Dr BALEN Frederic
Pole Médecine d'Urgence
CHU Toulouse
1 place Baylac
31058 Toulouse

Centre de Méthodologie et de Gestion des données :
Unité de Soutien Méthodologique à la Recherche (USMR) - CHU de Toulouse

Unité de vigilance de la recherche clinique :
Service de pharmacologie médicale et clinique & Direction de la Recherche, du Développement et de l'Innovation - CHU TOULOUSE

Ce protocole a été conçu et rédigé à partir de la version 4.0 du 18/01/2022 du protocole-type du GIRCI SOHO

HISTORIQUE DES MISES A JOUR DU PROTOCOLE

VERSION	DATE	RAISON DE LA MISE A JOUR
1.0	18/01/2024	Version soumise et refusée par le CPP SM IV
1.1	26/03/2024	Version déposée pour ré-examen par un nouveau CPP
1.2	05/06/2024	Version modifiée en réponse aux remarques du CPP
1.3	12/12/2024	Modification Substantielle MS1
1.4	10/03/2025	Version modifiée en réponse aux remarques du CPP

PAGE DE SIGNATURE DU PROTOCOLE

Impact d'une stratégie de prise en charge de la détresse respiratoire aiguë chez le sujet âgé basée sur l'utilisation de l'échographie cardiopulmonaire

LUC REED (LUng and Cardiac ultrasound for REspiratory Distress in ElDerly)

Code promoteur : RC31/23/0386

Promoteur

CHU Toulouse
Hôtel-Dieu,
2 rue Viguerie,
TSA 80035,
31059 Toulouse cedex

à Toulouse, le *:*

Titre et nom du représentant du promoteur
Signature :

Investigateur coordonnateur

Dr BALEN Frederic
Pole Médecine d'Urgence - CHU Toulouse
1 place Baylac - 31058 Toulouse
balen.f@chu-toulouse.fr

à Toulouse, le *:*

Titre et nom de l'investigateur
Signature :

Investigateur principal du centre

Titre Nom Prénom
Adresse
Mail

à XXXXXXXX, le :

Titre et nom de l'investigateur
Signature :

PRINCIPAUX CORRESPONDANTS

Investigateur coordonnateur
Dr BALEN Frederic
Pole Médecine d'Urgence
CHU Toulouse
1 place Baylac - 31058 Toulouse
balen.f@chu-toulouse.fr

Promoteur
CHU Toulouse, Hôtel-Dieu,
2 rue Viguerie,
TSA 80035,
31059 Toulouse cedex

Autres spécialités (Experts)
Dr DELMAS Clément
Soins Intensif de Cardiologie
CHU Toulouse
1 avenue Poulhes - 31400 Toulouse

Dr NOEL-SAVINA Elise
Soins Intensifs de Pneumologie
CHU Toulouse
24 chemin de Pouvourville - 31400 Toulouse

Dr DUBUCS Xavier
Pole Médecine d'Urgence - CHU Toulouse
1 place Baylac - 31058 Toulouse

Centre de Méthodologie et de Gestion des données
Dr Jason SHOURICK
Unité de Soutien Méthodologique à la Recherche (USMR)
CHU de Toulouse

Analyse Médico-économique
Dr Nadège COSTA
Unité DIM PMSI / Unité d'Evaluation médico-economique (UEME)
CHU de Toulouse

Coordination
HEBRARD Manon, ARC coordinatrice
Pole Médecine d'Urgence
CHU Toulouse
1 place Baylac - 31058 Toulouse

Unité de vigilance de la recherche clinique
Service de pharmacologie médicale et clinique

SOMMAIRE

Page de signature du protocole 3
SOMMAIRE **5**
1. RESUME DE LA RECHERCHE **8**
ABSTRACT **13**
2. JUSTIFICATION SCIENTIFIQUE ET DESCRIPTION GENERALE **14**
2.1. ETAT ACTUEL DES CONNAISSANCES 14
2.1.1. La dyspnée chez le sujet âgé 14
2.1.2. L'échographie clinique thoracique 14
2.2. HYPOTHESES DE LA RECHERCHE ET RESULTATS ATTENDUS 15
2.3. RAPPORT BENEFICE / RISQUE 15
2.4. RETOMBEES ATTENDUES 16
2.5. JUSTIFICATION DU FAIBLE NIVEAU D'INTERVENTION 16
3. OBJECTIFS DE LA RECHERCHE **17**
3.1. OBJECTIF PRINCIPAL 17
3.2. OBJECTIFS SECONDAIRES 17
4. CRITERES DE JUGEMENT **18**
4.1. CRITERE DE JUGEMENT PRINCIPAL 18
4.2. CRITERES DE JUGEMENT SECONDAIRES 19
5. CONCEPTION DE LA RECHERCHE **20**
5.1. JUSTIFICATION DES CHOIX METHODOLOGIQUES 20
5.2. SCHEMA DE LA RECHERCHE 21
5.3. METHODES POUR LA RANDOMISATION 21
6. CRITERES D'ÉLIGIBILITE **22**
6.1. CRITERES D'INCLUSION 22
6.2. CRITERES DE NON INCLUSION 22
6.3. FAISABILITE ET MODALITES DE RECRUTEMENT 22
7. STRATEGIES DE LA RECHERCHE **24**
7.1. STRATEGIE EXPERIMENTALE : STRATEGIE ECHOGRAPHIE CLINIQUE 24
7.2. TRAITEMENT/STRATEGIE/PROCEDURE DE COMPARAISON 24
7.3. INSU 24
8. METHODOLOGIE DE L'EVALUATION MEDICO-ECONOMIQUE **25**
8.1. ANALYSE DES OUTCOMES CLINIQUES 25
8.2. ANALYSE DES COUTS 25
8.2.1. Recueil de la consommation de soin 25
8.2.2. Valorisation de la consommation de soins 26
8.2.2.1. COUTS DIRECTS MEDICAUX 26
8.2.2.2. COUTS DIRECTS NON MEDICAUX 27
8.2.2.3. COUT DE PRODUCTION DE LA MISE EN ŒUVRE DE L'ECHOGRAPHIE PULMONAIRE ET CARDIAQUE 27
8.3. ANALYSES D'EFFICIENCE 27
8.4. ANALYSE D'IMPACT BUDGETAIRE 28
9. DEROULEMENT DE LA RECHERCHE **29**

9.1. CALENDRIER DE LA RECHERCHE 29
9.2. TABLEAU RECAPITULATIF DU SUIVI PARTICIPANT 29
9.3. VISITE D'INCLUSION 29
9.3.1. Recueil du consentement 29
9.3.2. Déroulement de la visite 30
9.4. VISITES DE SORTIE DES URGENCES 31
9.5. VISITE DE FIN DE LA RECHERCHE 31
9.6. REGLES D'ARRET DE LA PARTICIPATION D'UNE PERSONNE A LA RECHERCHE 32
9.7. CONTRAINTES LIEES A LA RECHERCHE ET INDEMNISATION EVENTUELLE DES PARTICIPANTS 32

10. GESTION DES ÉVÉNEMENTS INDÉSIRABLES / EFFETS INDESIRABLES / INCIDENTS 33

11. ASPECTS STATISTIQUES 34

11.1. CALCUL DE LA TAILLE D'ETUDE 34
11.2. METHODES STATISTIQUES EMPLOYEES 35
11.3. METHODES STATISTIQUES EMPLOYEES POUR LA PARTIE ECONOMIQUE 36

12. GESTION ET TRAITEMENT DES DONNEES ET DOCUMENTS SOURCE 37

12.1. ACCES AUX DONNEES 37
12.2. DONNEES SOURCE 37
12.2.1. Données issues du SNDS 37
12.3. CONFIDENTIALITE DES DONNEES 38

13. CONTROLE ET ASSURANCE QUALITE 40

13.1. CONSIGNES POUR LE RECUEIL DES DONNEES 40
13.2. CONTROLE QUALITE 40
13.3. GESTION DES DONNEES 40
13.4. AUDIT ET INSPECTION 41

14. CONSIDERATIONS ETHIQUES ET REGLEMENTAIRES 41

15. CONSERVATION DES DOCUMENTS ET DES DONNEES RELATIFS A LA RECHERCHE 43

16. RAPPORT FINAL 45

17. REGLES RELATIVES A LA PUBLICATION 45

17.1. COMMUNICATION DES RESULTATS AUX PARTICIPANTS 46
17.2. CESSION DES DONNEES 46

REFERENCES BIBLIOGRAPHIQUES ERREUR ! SIGNET NON DEFINI.

ANNEXE 1 : EVALUATION CLINICO-ECHOGRAPHIQUE DU PATIENT AGE EN DETRESSE RESPIRATOIRE 49

ANNEXE 2 : DOSSIER EXPERT 50

ANNEXE 3 : EQ-5D-5L 54

ANNEXE 4: QUESTIONNAIRE MEDICO-ECONOMIQUE 56

LISTE DES ABREVIATIONS

ANSM	Agence Nationale de Sécurité du Médicament et des produits de santé
BPCO	Bronchopneumopathie Chronique obstructive
CPP	Comité de Protection des Personnes
EvI	Evènement Indésirable
EvIG	Evènement Indésirable Grave
EIG	Effet Indésirable Grave
OAP	Œdème Aigu du Poumon
SU	Structure d'Urgence

1. RESUME DE LA RECHERCHE

**PROMOTEUR**	CHU Toulouse
**INVESTIGATEUR COORDONNATEUR/PRINCIPAL**	Dr BALEN Frederic<br>Pole Médecine d'Urgence - CHU Toulouse<br>balen.f@chu-toulouse.fr
**TITRE**	LUC REED (LUng and Cardiac ultrasound for REspiratory Distress in ElDerly)<br>Impact d'une stratégie de prise en charge de la détresse respiratoire aiguë chez le sujet âgé basée sur l'utilisation de l'échographie cardiopulmonaire
**JUSTIFICATION / CONTEXTE**	La dyspnée aigue est un motif fréquent et grave de recours aux Structures d'Urgence (SU), avec une mortalité à 1 mois proche de 16%. Elle pose la difficulté du diagnostic à la face initiale d'évaluation car l'origine de ce symptôme peut être variée (cardiologique, pulmonaire, infectieuse…) et la symptomatologie trompeuse. Cette difficulté diagnostique retarde la mise en place d'une prise en charge thérapeutique adaptée, alors même que la précocité de la prise en charge est associée avec une réduction de la mortalité. Ces problématiques sont particulièrement importantes chez le sujet âgé, chez qui l'erreur diagnostique et thérapeutique initiale ont une incidence estimée de 35% et entrainent une surmortalité importante (OR = 2,83).<br>Les examens complémentaires diagnostiques, en particulier biologiques, améliorent la prise en charge et le devenir des malades dyspnéiques aux urgences. Cependant ils ne sont pas disponibles immédiatement, incitant le clinicien à initier des traitements sans attendre leur(s) résultat(s), ce qui ne résout donc pas la problématique des traitements inappropriés.<br>L'échographie pulmonaire réalisée par le médecin urgentiste, disponible immédiatement au lit du patient, pourrait permettre de réduire le retard diagnostique et donc thérapeutique. Ses performances diagnostiques semblent excellentes, avec une AUC (Aire Under Curve ROC) de 0,95 pour la pneumopathie, de 0,91 pour l'Œdème Aigu du Poumon (OAP) et de 0,91 pour la décompensation de Broncho-Pneumopathie Chronique obstructive (BPCO). L'association à l'échographie pulmonaire d'une évaluation hémodynamique améliorerait ces performances. Ce protocole d'échographie clinique thoracique (LuCUS protocole pour « Lung and Cardiac UltraSound ») associe une évaluation d'échographie pulmonaire et une échocardiographie d'urgence (évaluation de la fonction systolique cardiaque visuelle et doppler

	transmitral et tissulaire à l'anneaux mitral). Il permettrait de réduire de 50% les thérapeutiques inappropriées.<br>Cependant, l'impact sur la prise en charge thérapeutique d'une stratégie diagnostique basée sur l'échographie cardio-pulmonaire chez le sujet âgé dyspnéique n'a pas été évaluée. Les recommandations de bonne pratique de la SFMU concernant l'utilisation de l'échographie clinique en médecine d'urgence incitent à l'utilisation de cet outil mais sont donc de faible niveau de preuve. L'utilisation de l'échographie cardio-pulmonaire reste donc peu implémentée en pratique.<br>Notre hypothèse est que l'une stratégie diagnostique standardisée, basée sur l'utilisation de l'échographie pulmonaire et hémodynamique, permet une réduction des thérapeutiques inappropriées chez les patients âgés admis en SU pour dyspnée aigue.
**OBJECTIFS**	<u>Principal :</u> Evaluer l'impact sur l'inadéquation thérapeutique initiale d'une stratégie de prise en charge de la dyspnée aiguë chez le sujet âgé, basée sur l'utilisation de l'échographie cardiopulmonaire.<br><br><u>Secondaires :</u><br>- Evaluer l'impact d'une stratégie de prise en charge de la dyspnée aigue chez le sujet âgé, basée sur l'utilisation de l'échographie cardiopulmonaire, comparativement à une stratégie habituelle (soins courants), sur le parcours de soin des malades ainsi que sur la morbi-mortalité.<br>- Evaluer l'efficience de l'utilisation de l'échographie cardiopulmonaire en comparaison à la stratégie de prise en charge habituelle, au moyen d'analyses coût-utilité et coût-efficacité.<br>- Evaluer l'impact budgétaire de la mise en œuvre systématique de l'échographie cardiopulmonaire, du point de vue de l'hôpital et de l'assurance maladie.
**CRITERES DE JUGEMENT**	<u>Principal :</u> L'inadéquation thérapeutique à H1,définie en comparant le traitement initial aux urgences au diagnostic final après expertise.<br><br><u>Secondaires :</u><br>✓ Diagnostic correct à la sortie du SU<br>✓ Utilisation de l'échographie lors de la prise en charge du patient dans le bras contrôle<br>✓ Durée de passages aux urgences<br>✓ Durée d'hospitalisation post urgence : en médecine/chirurgie/obstétrique (MCO) ou soins de suite et de réadaptation (SSR)<br>✓ Nombre de jours en vie hors de l'hôpital entre J0 et J30<br>✓ Mortalité à J30

	Du point de vue économique, les critères d'évaluations pour calculer les ratios d'efficience à 30 jours sont :<br>- Les coûts directs médicaux et non médicaux, du point de vue de l'assurance maladie<br>- Les QALYs calculés au moyen du score d'utilité basé sur l'échelle EQ-5D-5L et la survie<br>- Le nombre de patients ayant bénéficié d'une prise en charge thérapeutique adaptée de manière précoce<br>En complément l'analyse d'impact budgétaire du point de vue de l'hôpital prendra en compte le coût de production de l'échographie pulmonaire ainsi que les coûts en lien avec la production des séjours hospitaliers. Du point de vue de l'AM, cette analyse prendra en compte les coûts directs médicaux et non médicaux.
**SCHEMA DE LA RECHERCHE**	Essai multicentrique interventionnel en stepped-wedge randomisé en cluster
**CRITERES D'INCLUSION**	- Patient de plus de 65 ans<br>- Admis en Service d'Urgence<br>- Pour dyspnée aiguë comme motif principal d'admission définie telle que :<br>- Sensation de souffle court ou de gêne respiratoire évoluant depuis moins de 2 semaines<br>- Signes objectifs de détresse respiratoire en préhospitalier ou à l'admission aux urgences :<br>✓ Polypnée ≥ 22 mouvements/min<br>✓ SpO2 ≤ 92% en air ambiant<br>- Patient pris en charge par un investigateur formé à l'échographie thoracique (DU, Formation WFF ou DES Médecine d'urgence)<br>- Inclusion après consentement du proche ou de la personne de confiance ou en son absence, par la procédure d'urgence et recueil du consentement éclairé a posteriori du patient ou, à défaut, du proche ou de la personne de confiance<br>- Patient affilié à un régime de sécurité sociale ou équivalent
**CRITERES DE NON INCLUSION**	- Dyspnée post traumatique<br>- Patients admis pour dyspnée sur COVID identifié (PCR réalisée en externe ou PCR rapide en SU)<br>- Détresse vitale nécessitant une intubation immédiate<br>- Fibrose pulmonaire évolutive<br>- Cancer pulmonaire ou métastatique au poumon connu avant l'inclusion<br>- Patient ayant bénéficié d'une prise en charge médicamenteuse spécifique à la dyspnée avant l'inclusion<br>- Patient identifié en fin de vie

	- Patient ayant déjà été inclus pour un épisode précédent<br>- Patient privé de liberté<br>- Patient sous tutelle, curatelle ou sauvegarde de justice
**TRAITEMENTS/STRATEGIES/PROCEDURES DE LA RECHERCHE**	La stratégie standard répondra aux protocoles et habitudes de services et le clinicien ne sera pas guidé dans ses prescriptions.<br>Lors de la phase « échographie clinique », les patients bénéficieront d'une échographie clinique suivant leur admission par le médecin urgentiste les prenant en charge, avant de débuter les thérapeutiques. Cette échographie comportera une évaluation pleuro-pulmonaire de l'ensemble des champs pulmonaires et une évaluation hémodynamique sur coupe cardiaque avec analyse du doppler transmitral. Un protocole thérapeutique est proposé selon les éléments cliniques et échographiques recueillis à l'admission. Le clinicien en charge du malade est libre de suivre les propositions faites. Une réévaluation avant la sortie du SU, lorsque les résultats biologiques et d'imagerie (radiographie et/ou scanner) seront disponibles, permettra d'engager de nouveaux traitements selon ces résultats et l'évolution du patient.<br>Du fait d'une randomisation en stepped wedge, clinicien et patients ne seront pas en aveugle de la procédure entreprise.<br>A la fin des inclusions, un comité d'adjudication (cardiologue et pneumologue) statuera sur le diagnostic final et l'adéquation thérapeutique sur dossier standardisé. Les experts seront aveugles du bras de randomisation lorsqu'ils détermineront le diagnostic final (ils ne seront pas mentionnés dans les dossiers d'expertise). Les dossiers d'expertise seront constitués par l'investigateur principal et l'ARC du projet afin de proposer un dossier d'expertise standardisé.
**TAILLE D'ETUDE**	504 patients (252 dans chaque bras)
**DUREE DE LA RECHERCHE**	Durée de la période d'inclusion : 24 mois<br>Durée de participation de chaque participant : 1 mois<br>Durée totale de la recherche : 25 mois
**ANALYSE STATISTIQUE DES DONNEES**	L'analyse sera réalisée en intention de traiter. Pour l'analyse du critère de jugement principal, un modèle à effet mixte sera appliqué pour comparer l'inadéquation thérapeutique initiale entre les deux stratégies comparées, à l'aide d'une régression logistique à effet mixte incluant l'effet centre comme intercept aléatoire. Ce modèle sera ajusté sur la période de recrutement (prise en compte comme facteur à effet fixe). Une interaction centre*temps sera recherchée en testant un effet « pente aléatoire » du délai entre le début de l'étude et la date de

	recrutement. Le modèle sera ajusté sur les facteurs de confusion potentiels, notamment âge, sexe, et antécédents (cardiopathie, respiratoire, rénal, diabète démence).<br>Concernant l'étude économique, des analyses de sensibilité déterministe et probabiliste seront réalisées. L'analyse de sensibilité probabiliste, sera réalisée à l'aide de la méthode du bootstrap non-paramétrique. Elle nous permettra d'identifier l'incertitude autour de l'ICER en estimant son intervalle de confiance. De plus, une courbe d'acceptabilité sera construite. Elle permet d'apprécier, de manière visuelle, la relation entre l'incertitude du ratio coût-utilité différentiel et la valeur seuil consentit par le payeur (i.e. Disposition à Payer Collective).
**RETOMBEES ATTENDUES**	La vérification de l'hypothèse permettrait de prouver le bénéficie réel au patient dyspnéique d'une stratégie standardisée d'évaluation initiale et précoce par échographie clinique (disponible immédiatement au lit du patient). Bien que ce bénéficie soit fortement suspecté, il n'est pas prouvé à l'heure actuelle.<br>Cela pourrait argumenter un changement des pratiques et convaincre la communauté des urgentistes de son efficacité en pratique quotidienne. Il faudrait donc considérer la formation de l'ensemble des urgentistes à l'échographie pulmonaire et son utilisation chez le patient dyspnéique.

ABSTRACT

This research has been registered in http://www.clinicaltrials.gov/.

Impact of a management strategy for acute dyspnea in elderly subjects based on the use of lung and cardiac ultrasonography

LUC REED (LUng and Cardiac ultrasound for REspiratory Distress in ElDerly)

University Hospital Toulouse is the sponsor of this research.
This research will be conducted with the support of *PHRCI*

- **Brief summary :** Prospective trial to evaluate the impact on the initial therapeutic inadequacy of a management strategy for acute dyspnea in the elderly based on the use of lung and cardiac ultrasonography.
- **Detailed description :** Acute dyspnea is a frequent and serious reason of admission in Emergency Department (ED), with a one-month mortality close to 16%. It is difficult to diagnose in the initial assessment phase since the cause of this symptom can vary (cardiological, pulmonary, infectious, etc.) and the symptoms can be misleading. This difficulty in diagnosing delays the implementation of appropriate therapeutic management even as the timeliness of management is associated with a reduction in mortality. These issues are particularly important in the elderly. Lung and cardiac ultrasonography performed by the emergency physician, immediately available at the patient's bedside, could reduce the diagnostic and therefore therapeutic delay. However, the impact of a diagnostic strategy based on lung and cardiac ultrasonography in dyspneic elderly subjects has not been evaluated.
  We propose to randomize patients in two groups : "standard of care" or "clinical ultrasound" group. Treatments initiated in ED will be noted to be compared to final diagnosis.
- **Primary outcome:** The principal outcome is therapeutic inadequacy between initiated emergency treatments and the final diagnosis made by expert opinion.
- **Study design :** Prospective, stepped-wedge randomized, multicenter (inter-regional), interventional study aiming to evaluate the clinical impact of a diagnostic tool.
- **Eligibility criteria:**
  - Inclusion criteria: Patient over 65 years of age admitted to ED for acute dyspnea as chief complaint (defined as: sensation of shortness of breath progressing for less than 2 weeks) with objective signs of respiratory distress (Polypnea $\geq$ 22 breaths/min and $SpO_2 \leq 92\%$ on room air), consent to participate in the study or, failing that, a close relative or trusted support person, with the possibility of inclusion via the emergency procedure and the collection of informed consent a posteriori.
  - Exclusion criteria: patient identified at end of life or patient already included
- **Arm number or label and arm type** : The "clinical ultrasound" group will be subject to a diagnostic strategy based on the protocolized implementation of clinical lung and cardiac ultrasonography, with a proposed diagnostic and therapeutic focus based on the results.

  The "standard care" group will be subject to a diagnostic and therapeutic strategy based on the usual practices of the department and the clinician.
- **Number of subjects :** 504 patients
- **Statistical analysis :** Generalized linear regression models
- **Conditions :** Elderly, Point-of-Care Ultrasound, Respiratory Distress Syndrom

2. JUSTIFICATION SCIENTIFIQUE ET DESCRIPTION GENERALE

2.1. ETAT ACTUEL DES CONNAISSANCES

2.1.1. LA DYSPNEE CHEZ LE SUJET AGE

La dyspnée aiguë est un motif fréquent de recours aux Structures d'Urgence (SU) adultes, représentant près de 5% de nos admissions hors épidémie. Sa mortalité intra-hospitalière est élevée, proche de 16% (1). Elle pose la difficulté du diagnostic à la phase initiale d'évaluation car l'origine de ce symptôme peut être variée (cardiologique, pulmonaire, infectieuse…) (2–4). Il n'existe pas de traitement unique à la dyspnée car le traitement dépend du diagnostic responsable de la dyspnée. L'œdème aigu du poumon (OAP) doit être traité par diurétiques et dérivés nitrés (5), la pneumopathie bactérienne par antibiotique (6), la décompensation de Bronchopneumonie Chronique Obstructive (BPCO) par β2-mimétiques (7) et l'embolie pulmonaire par anticoagulant (8). La symptomatologie est trompeuse (9), particulièrement chez le sujet âgé, rendant la démarche diagnostique initiale particulièrement complexe (1). Cette difficulté diagnostique retarde la mise en place des thérapeutiques adaptées, alors même que la précocité de la prise en charge est associée à une réduction de la mortalité (10,11). Ces problématiques sont particulièrement importantes chez le sujet âgé (12), chez qui l'erreur diagnostique et l'inadéquation thérapeutique initiale ont une incidence estimée de 35% et entrainent une surmortalité importante (OR = 2,83) en cas de détresse respiratoire(1). La détresse respiratoire est en effet « le stade ultime » de la dyspnée, associant la sensation subjective de « difficulté respiratoire » à des signes cliniques objectifs (polypnée, balancement, et éventuelle désaturation).

Les examens complémentaires diagnostiques, en particulier biologiques, améliorent la prise en charge et le devenir des malades dyspnéiques aux urgences (13,14). Cependant ils ne sont pas disponibles immédiatement, incitant le clinicien à initier des traitements sans attendre leur(s) résultat(s), ce qui ne résout donc pas la problématique des traitements inappropriés.

2.1.2. L'ECHOGRAPHIE CLINIQUE THORACIQUE

L'échographie pulmonaire réalisée par le médecin urgentiste, disponible immédiatement au lit du patient, pourrait permettre de réduire le retard diagnostique et donc thérapeutique. Ses performances diagnostiques semblent excellentes (15), avec une AUC (Aire Under Curve ROC) de 0,95 pour la pneumopathie, de 0,91 pour l'Œdème Aigu du Poumon (OAP) et de 0,91 pour la décompensation de Broncho-Pneumopathie Chronique Obstructive (BPCO). L'association, à l'échographie pulmonaire, d'une évaluation hémodynamique améliorerait ces performances (16). Ce protocole d'échographie clinique thoracique (LuCUS protocole pour « Lung and Cardiac UltraSound ») associe une évaluation d'échographie pulmonaire et une échocardiographie d'urgence (évaluation de la fonction systolique cardiaque visuelle et doppler transmitral et tissulaire à l'anneau

mitral). Une stratégie combinant échographie pulmonaire et cardiaque dans la démarche diagnostique de la dyspnée du sujet âgé admis en SU a récemment prouvé sa performance diagnostique (17,18) mais son impact sur la qualité de prise en charge des malades n'est pas encore démontré. Elle permettrait de réduire de 50% les thérapeutiques inappropriées (16). Les recommandations de bonne pratique de la Société Française de Médecine d'Urgence (SFMU) (19) concernant l'utilisation de l'échographie clinique en médecine d'urgence incitent à l'utilisation de cet outil mais sont de faible niveau de preuve. L'utilisation de l'échographie cardio-pulmonaire reste donc peu implémentée en pratique.

2.2. HYPOTHESES DE LA RECHERCHE ET RESULTATS ATTENDUS

Notre hypothèse est qu'une stratégie diagnostique standardisée, basée sur l'utilisation de l'échographie pulmonaire et cardiaque, permet une réduction des thérapeutiques inappropriées chez les patients âgés admis en SU pour dyspnée aiguë. Cette hypothèse semble licite : des travaux récents démontre une diminution de près de 15% en population dyspnéique aux urgences (20,21). Cependant il s'agit de cohortes de faible effectif et avec un population jeune (67 ans d'âge moyen), chez qui l'inadéquation thérapeutique est peu fréquente.

En population âgée, nous attendons une réduction de 50% (16) de l'inadéquation thérapeutique dans le groupe « échographie clinique thoracique » comparée au groupe « standard » (soit une inadéquation thérapeutique de 17,5% *vs* 35%). En effet notre équipe à récemment démontré les bonnes performances de l'échographie cardio-pulmonaire chez le sujet âgé lors de l'étude ECHOP (18).

D'un point de vue économique, l'hypothèse repose sur le fait que la stratégie de santé basée sur l'utilisation de l'échographie pulmonaire et cardiaque permet une amélioration de l'état de santé en termes d'initiation de thérapeutique adaptée et de qualité de vie ainsi qu'une réduction des dépenses de santé à 1 mois en lien avec une réduction en termes de délai de l'errance diagnostique et une prise en charge adaptée plus précoce. En complément, nous supposons que les déterminants socio-économiques, tels que le niveau de revenu, le niveau d'éducation et la catégorie socio-professionnelle pourraient avoir un impact sur l'efficacité, les coûts de prise en charge et l'efficience en lien avec l'utilisation de l'échographie pulmonaire et cardiaque.

2.3. RAPPORT BENEFICE / RISQUE

Les risques décrits de l'utilisation de l'échographie sont obstétricaux ou optiques, et ne concernent donc pas notre population d'étude.

Les bénéfices attendus sont une diminution des thérapeutiques inadaptées pour les patients bénéficiant d'une échographie cardio-pulmonaire lors de leur passage en SU pour dyspnée.

Dans le groupe « standard », le praticien sera libre d'utiliser l'échographie (non guidée) s'il le juge utile, selon ses pratiques habituelles.

La balance semble largement en faveur du bénéfice mais reste à démontrer.

2.4. RETOMBEES ATTENDUES

La vérification de l'hypothèse permettrait de prouver le bénéficie réel (meilleure adéquation thérapeutique) au patient dyspnéique d'une stratégie standardisée d'évaluation initiale et précoce par échographie clinique. Bien que ce bénéficie soit fortement suspecté, il n'est pas prouvé à l'heure actuelle.

Cela pourrait argumenter un changement des pratiques. Il faudrait donc considérer la formation de l'ensemble des urgentistes à l'échographie cardio-pulmonaire et son utilisation chez le patient dyspnéique.

2.5. JUSTIFICATION DU FAIBLE NIVEAU D'INTERVENTION

L'utilisation de l'échographie n'est pas irradiante et réalisable au lit du malade en complément de l'examen clinique. Elle fait l'objet de recommandation de faible niveau de preuve dans l'indication (19), ce qui rend son utilisation en pratique peu fréquente à l'heure actuelle (moins de 10% des patients dyspnéiques admis en SU).

3. OBJECTIFS DE LA RECHERCHE

3.1. OBJECTIF PRINCIPAL

Evaluer l'impact d'une stratégie de prise en charge de la dyspnée aiguë chez le sujet âgé, basée sur l'utilisation de l'échographie cardiopulmonaire, sur la réduction de l'inadéquation thérapeutique initiale comparativement à une stratégie habituelle (soins courants).

3.2. OBJECTIFS SECONDAIRES

Evaluer l'impact d'une stratégie de prise en charge de la dyspnée aiguë chez le sujet âgé, basée sur l'utilisation de l'échographie cardiopulmonaire, comparativement à une stratégie habituelle (soins courants), sur :

- La prise en charge, évaluée par :
    - le taux de diagnostics corrects après évaluation initiale et en sortie des urgences
    - la proportion d'utilisation de l'échographie
    - la durée de passages aux urgences
    - la durée d'hospitalisation
- La morbi-mortalité, évaluée par :
    - le taux de ré-hospitalisation
    - le nombre de jours en vie hors de l'hôpital entre J0 et J30
    - la mortalité à J30

D'un point de vue économique, les objectifs sont :

Evaluer l'efficience de l'utilisation de l'échographie cardio-pulmonaire en comparaison à la stratégie de prise en charge habituelle, au moyen d'analyses cout-utilité et cout-efficacité.

Evaluer l'impact budgétaire de la mise en œuvre systématique de l'échographie cardio-pulmonaire, du point de vue de l'hôpital et de l'assurance maladie.

Mesurer l'effet du statut socio-économique sur les coûts de prise en charge, l'efficacité et l'efficience de l'utilisation de l'échographie pulmonaire et cardiaque pour le diagnostic des dyspnées chez le sujet âgé.

4. CRITERES DE JUGEMENT

4.1. CRITERE DE JUGEMENT PRINCIPAL

L'inadéquation thérapeutique est définie à partir du traitement initial (H1) aux urgences comparé au diagnostic final après expertise. Sont considérés comme inadéquats :

✓Chez un patient en OAP :
- La prescription de B2-mimétiques ou d'antibiotique (hors cause septique)
- La non-prescription de diurétique et de dérivés nitrés si TAS > 140 mmHg

✓Chez un patient présentant une décompensation de BPCO :
- La prescription de diurétiques ou de dérivés nitrés
- La non-prescription de B2-mimétiques

✓Chez un patient présentant une crise d'asthme :
- La prescription de diurétiques ou de dérivés nitrés
- La non-prescription de B2-mimétiques et de corticoides

✓Chez un patient ayant une infection pulmonaire :
- La prescription de diurétiques, de dérivés nitrés ou de B2-mimétique
- La non-prescription d'antibiotique

✓Chez le patient présentant une embolie pulmonaire :
- La prescription de diurétiques, de dérivés nitrés ou de B2-mimétique
- La non-prescription d'anticoagulation à dose curative

En cas de diagnostic « Autre », le traitement adapté sera jugé lors de l'expertise.

En cas de diagnostic associés, les thérapeutiques inadaptés seront celles n'étant pas recommandées dans les diagnostics retenus.

Le classement diagnostique (OAP, Décompensation de BPCO, crise d'asthme, Infection Pulmonaire, Embolie pulmonaire, autre ou une association de diagnostics) et l'adéquation/inadéquation thérapeutique seront établis par expertise des dossiers par 2 experts (un cardiologue (CD) et un pneumologue (ENS)) aveugles des résultats de l'échographie clinique et de l'autre expert. L'expertise sera centralisée sur le CHU de Toulouse et réalisée par les experts associés au projet. En cas de discordance entre les deux experts, un troisième expert (urgentiste formé à la prise en charge des patients gériatrique (XD)) sera sollicité. Les dossiers d'expertise mis à disposition des experts comprendront les données recueillies lors du passage aux urgences. Les dossiers d'expertise (Annexe 2) comprendront : les variables démographiques (âge, sexe, antécédents cardiologiques, respiratoires, diabète ou rénaux), les paramètres d'entrée (tension artérielle, fréquence cardiaque, fréquence respiratoire, saturation en oxygène, température), des données d'examen clinique (auscultation pulmonaire, auscultation cardiaque, présence d'œdème des membres inférieurs ou de turgescence jugulaire), l'ECG d'entrée, les principaux résultats

biologiques (gazométrie artérielle, NT-Pro-BNP, hémoglobine, globules blancs, CRP) et radiologiques réalisés (radiographie ou TDM). Les données de l'échographie clinique ne seront pas disponibles pour maintenir l'aveugle.

4.2. CRITERES DE JUGEMENT SECONDAIRES

- Diagnostic correct après évaluation initiale et à la sortie du SU, comparé au diagnostic final après expertise
- Utilisation de l'échographie lors de la prise en charge du patient dans le bras contrôle
- Durée de passages aux urgences en heures
- Durée d'hospitalisation post urgence : en médecine/chirurgie/obstétrique (MCO) ou soins de suite et de réadaptation (SSR)
- Nombre de jours en vie hors de l'hôpital entre J0 et J30
- Mortalité à J30

Du point de vue économique, les critères d'évaluations pour calculer les ratios d'efficience à 30 jours sont :

- Les coûts directs médicaux et non médicaux, du point de vue de l'assurance maladie
- Les QALYs calculés au moyen du score d'utilité basé sur l'échelle EQ-5D-5L et la survie
- Le nombre de patients ayant bénéficié d'une prise en charge thérapeutique adaptée de manière précoce

En complément l'analyse d'impact budgétaire du point de vue de l'hôpital prendra en compte le coût de production de l'échographie pulmonaire ainsi que les coûts en lien avec la production des séjours hospitaliers. Du point de vue de l'AM, cette analyse prendra en compte les coûts directs médicaux et non médicaux.

Enfin, le niveau de revenu, le niveau d'éducation et la catégorie socio-professionnelle (avant le passage à la retraite) seront recueillis afin de définir le statut socio-économique du patient.

5. CONCEPTION DE LA RECHERCHE

5.1. JUSTIFICATION DES CHOIX METHODOLOGIQUES

La population cible qui bénéficierait le plus d'une évaluation échographique précoce sont les patients admis pour une dyspnée « d'origine indéterminée » lors de l'examen initial. Nous choisissons donc de cibler une population âgée, chez qui le diagnostic initial est particulièrement complexe. L'utilisation de score clinique pré-test aurait pu être envisagée afin d'inclure des patients a probabilités intermédiaires d'OAP, BPCO ou pneumopathie. Cependant ce choix ne semble pas pragmatique car obligerait à calculer les scores pré-test spécifiques à chaque pathologie évoquée.

De même les patients avec un diagnostic de COVID seront exclus. En effet ces patients sont souvent admis en SU avec des test PCR déjà positifs ou bénéficient de PCR rapide dès leur admission, simplifiant grandement la démarche diagnostique et rendant l'intérêt de l'utilisation de l'échographie limitée.

Nous faisons le choix pragmatique de réaliser une étude en stepped wedge. En effet, la randomisation informatisée en urgence est un frein majeur aux inclusions. L'essai se déroule aux urgences où le flux des patients est constant. Nous nous intéressons, de plus, à une symptomatologie qui peut s'avérer grave, une randomisation au niveau du patient pourrait donc être un obstacle à l'inclusion des patients. De plus en modifiant le parcours de soins, le temps passé au lit du patient et les examens complémentaires prescrits, l'utilisation de l'échographie est susceptible d'avoir des effets sur l'ensemble de l'unité et la randomisation individuelle serait donc inadaptée.

D'autre part, le choix du stepped wedge permet le rappel du protocole et rappel des mesures échographiques attendues dans chaque centre lors du passage en phase interventionnelle (stratégie échographie thoracique). C'est le praticien en charge du patient qui inclura le patient, le prendra en charge, et réalisera l'échographie lors de la phase intervention. Nous assumons de ne pas faire réaliser l'échographie par un « expert » afin d'apporter des données plus représentatives de la pratique courante en SU. En phase intervention, les thérapeutiques seront guidées par une proposition basée sur les résultats clinico-échographiques, afin de limiter le biais d'interprétation de l'échographie. L'adéquation thérapeutique sera jugée par rapport au diagnostic final expertisé sur dossier par un cardiologue et un pneumologue, en l'absence de gold standard unique pour le diagnostic de l'ensemble des pathologies considérées. Cette évaluation du critère de jugement par les deux experts se fera sur dossier standardisé, en aveugle de la stratégie de prise en charge pour limiter les biais liés à l'absence de double aveugle au moment de la prise en charge.

5.2. SCHEMA DE LA RECHERCHE

L'étude LUC-REED est une étude multicentrique, interventionnelle, randomisée en cluster de type stepped-wedge.

5.3. METHODES POUR LA RANDOMISATION

La randomisation est assurée par un schéma d'étude en stepped-wedge, en cluster. Les unités de randomisation sont les centres hospitaliers, au nombre de sept. Du fait du nombre limité d'unités à randomiser, il n'est pas prévu de stratifier cette randomisation.

L'étude se déroulera sur 8 périodes de 3 mois (pour une durée totale de 24 mois) :

- au début la prise en charge est la stratégie habituelle (notée A), tous les centres commencent avec cette stratégie habituelle ;
- puis à un temps différent pour chaque séquence (3 mois, 6 mois, 9 mois, 12 mois, 15 mois, 18 mois, 21 mois), la prise en charge bascule en une stratégie basée sur l'utilisation de l'échographie cardiopulmonaire (notée B).

Une séquence sera attribuée aléatoirement à chaque centre. La liste de randomisation sera générée par l'Unité de Soutien Méthodologique à la Recherche clinique (USMR), avant le début d'étude et communiquée aux investigateurs des différents centres pour qu'ils puissent anticiper les formations et temps de bascules.

Ce calendrier permet d'obtenir autant de périodes hivernales (en bleu) dans chaque groupe, afin de limiter l'effet de la saisonnalité sur le recrutement dans chaque bras.

<u>Tableau : 1 : Séquences de randomisation</u>

	Sept - Oct A1		Nov - Dec A1		Jan - Fev A1		Mars - Avr A1		Mai - Juin A1		Juil - Aout A1		Sept - Oct A2		Nov - Dec A2		Jan - Fev A2		Mars - Avr A2		Mai - Juin A2		Juil - Aout A2	
Centre 1	A	A	A	B	B	B	B	B	B	B	B	B	B	B	B	B	B	B	B	B	B	B	B	B
Centre 2	A	A	A	A	A	A	B	B	B	B	B	B	B	B	B	B	B	B	B	B	B	B	B	B
Centre 3	A	A	A	A	A	A	A	A	A	B	B	B	B	B	B	B	B	B	B	B	B	B	B	B
Centre 4	A	A	A	A	A	A	A	A	A	A	A	A	B	B	B	B	B	B	B	B	B	B	B	B
Centre 5	A	A	A	A	A	A	A	A	A	A	A	A	A	A	A	B	B	B	B	B	B	B	B	B
Centre 6	A	A	A	A	A	A	A	A	A	A	A	A	A	A	A	A	A	A	B	B	B	B	B	B
Centre 7	A	A	A	A	A	A	A	A	A	A	A	A	A	A	A	A	A	A	A	A	A	B	B	B

Exposition A= soins courants ; Exposition B = utilisation de l'échographie cardiopulmonaire

Les investigateurs et les patients ne seront pas en aveugle. Seule l'évaluation du critère de jugement principal par les experts se fera en aveugle du type de prise en charge.

6. CRITERES D'ÉLIGIBILITE

6.1. CRITERES D'INCLUSION

- Patient de plus de 65 ans
- Admis en Service d'Urgence
- Pour dyspnée aiguë comme motif principal d'admission définie telle que :
    - Sensation de souffle court ou de gêne respiratoire évoluant depuis moins de 2 semaines
    - Signes objectifs de détresse respiratoire en préhospitalier ou à l'admission aux urgences :
        - ✓ Polypnée ≥ 22 mouvements/min
        - ✓ SpO2 ≤ 92% en air ambiant
- Pris en charge par un investigateur formé à l'échographie thoracique (DU, Formation WFF ou DES Médecine d'urgence)
- Inclusion après consentement du proche ou de la personne de confiance ou en son absence, par la procédure d'urgence et recueil du consentement libre et éclairé a posteriori du patient ou, à défaut, du proche ou de la personne de confiance
- Patient affilié à un régime de sécurité sociale ou équivalent

6.2. CRITERES DE NON INCLUSION

- Dyspnée post traumatique
- Patients admis pour dyspnée sur COVID identifié (PCR réalisée en externe ou PCR rapide en SU)
- Détresse vitale nécessitant une intubation immédiate
- Fibrose pulmonaire évolutive
- Cancer pulmonaire ou métastatique au poumon connu avant l'inclusion
- Patient ayant bénéficié d'une prise en charge médicamenteuse spécifique à la dyspnée avant l'inclusion
- Patient identifié en fin de vie
- Patient ayant déjà été inclus pour un épisode précédent
- Patient privé de liberté
- Patient sous tutelle, curatelle ou sauvegarde de justice

6.3. FAISABILITE ET MODALITES DE RECRUTEMENT

Les patients éligibles seront recrutés à leur admission en SU. La dyspnée représente 5% des motifs de consultation aux urgences, majoritairement de sujets âgés, soit environ 150 patients admis pour dyspnée par centre par mois. Le recrutement de 3 patients /centre/mois semble réalisable.

Comme la situation d'urgence ne permet pas de recueillir le consentement du patient, celui d'un membre de sa famille ou celui de la personne de confiance sera recherché s'ils sont présents. Dans les autres cas, l'inclusion sera réalisée dans le contexte de l'urgence et l'intéressé ou, le cas échéant, les membres de la famille ou la personne de confiance sont informés dès que possible et leur consentement leur sera demandé pour la poursuite éventuelle de cette recherche.

Le plan expérimental en stepped wedge assure de pouvoir réaliser une « mise à niveau » des urgentistes de chaque centre pour la réalisation de l'échographie cardiaque et pulmonaire. De plus, la majorité des urgentistes des centres inclueurs sont déjà formés à la technique.

7. STRATEGIES DE LA RECHERCHE

7.1. STRATEGIE EXPERIMENTALE : STRATEGIE ECHOGRAPHIE CLINIQUE

Lors de la phase « échographie clinique », les patients bénéficieront d'une échographie clinique suivant leur admission par le médecin urgentiste les prenant en charge, avant de débuter les thérapeutiques. Une demi-journée de rappel au protocole aura été proposée dans chaque centre avant de basculer dans le bras « échographie clinique ». Cette échographie comportera une évaluation pleuro-pulmonaire de l'ensemble des 6 champs pulmonaires et une évaluation hémodynamique sur coupe cardiaque avec analyse du doppler transmitral. Le choix des profils pulmonaire associés aux différents diagnostics sont justifiés par les performances de cette dernière (15). Les choix des cut-of des doppler transmitraux et tissulaires sont ceux proposés par des étude pragmatiques en médecine d'urgence (16,22). L'investigateur sera libre du choix de l'échographe et des type de sondes utilisées, selon le matériel disponible dans le service. Un protocole thérapeutique est proposé selon les éléments cliniques et échographiques recueillis à l'admission (Annexe 1). Le clinicien en charge du malade est libre de suivre les propositions faites. Une réévaluation avant la sortie du SU, lorsque les résultats biologiques et d'imagerie (radiographie et/ou scanner) seront disponibles, permettra d'engager de nouveaux traitements selon ces résultats et l'évolution du patient.

7.2. TRAITEMENT/STRATEGIE/PROCEDURE DE COMPARAISON

La stratégie standard répondra aux protocoles et habitudes de services et le clinicien ne sera pas guidé dans ses prescriptions. Le clinicien en charge du patient sera donc libre d'utiliser l'échographie, non guidée, s'il le juge nécessaire. La réalisation éventuelle d'une échographie sera relevée.

7.3. INSU

Du fait d'une randomisation en stepped wedge, clinicien et patients ne seront pas aveugles de la procédure entreprise.

Les dossiers d'expertise seront accessibles via l'eCRF à partir des données saisies par les centres mais les experts n'auront accès qu'aux données du dossier d'expertise standardisé (Décrit en Annexe 2). Les experts seront donc en aveugle de la procédure choisie et des traitements initiés aux urgences lorsqu'ils détermineront le diagnostic final (ces données ne seront pas accessibles aux experts dans l'eCRF).

8. METHODOLOGIE DE L'EVALUATION MEDICO-ECONOMIQUE

L'analyse médico-économique dans le cadre de ce projet consistera d'une part en des analyses d'efficience du point de vue de l'assurance maladie, comparant l'utilisation de l'échographie pulmonaire et cardiaque pour le dépistage de la dyspnée chez le sujet âgé à la prise en charge conventionnelle et d'autre part, à une analyse d'impact budgétaire du point de vue de l'assurance maladie et des établissements hospitaliers. En complément, une analyse de l'impact des caractéristiques socio-économiques sur l'efficacité les coûts de prise en charge et l'efficience de l'utilisation de l'échographie pulmonaire et cardiaque pour le diagnostic de la dyspnée chez le sujet âgé, sera effectuée.

8.1. ANALYSE DES OUTCOMES CLINIQUES

L'analyse coût-utilité constituera notre analyse principale. Le critère d'utilité sera la survie pondérée par la qualité de vie des patients à 1 mois (QALY). L'estimation de la qualité de vie sera mesurée au moyen de l'échelle EuroQol-5D-5L (ANNEXE 3). L'EQ-5D-5L est un questionnaire auto-administré, générique et multidimensionnel (23). C'est la version améliorée de l'EQ-5D 3L. La sensibilité de l'EQ-5D est améliorée et les effets seuils ont été réduits grâce à l'augmentation du nombre de niveaux (c.-à-d. de 3 à 5). Il comporte deux composantes, une composante descriptive et une échelle visuelle analogique (EQ-VAS). La composante descriptive est composée de 5 dimensions décrites par 5 niveaux qui permettent de définir l'état de santé. L'EQ-5D-5L est traduit, validé en français et dispose d'une fonction d'utilité. Cette fonction d'utilité est calculée sur la base des préférences révélées par la population française (24).
L'analyse de l'efficacité reposera sur le nombre de patient pour lesquels une prise en charge thérapeutique adaptée de manière précoce aura été mise en œuvre.

8.2. ANALYSE DES COUTS

8.2.1. RECUEIL DE LA CONSOMMATION DE SOIN

L'évaluation des coûts sera réalisée du point de vue de l'assurance maladie (HAS 2020) . Les dépenses occasionnées par la prise en charge des patients dans chaque bras (en lien ou non avec le diagnostic et le traitement de la dyspnée), seront comptabilisées sur une période de 1 mois. Une mesure des quantités physiques des ressources consommées et une valorisation monétaire de ces ressources seront réalisées.

Le tableau ci-dessous présente les données de nature économique qui seront recueillies au cours de l'étude .

Coûts directs médicaux	Coûts directs non médicaux
Hospitalisation : MCO, psychiatrique, SSR<br>Soins ambulatoires : consultations, actes médicaux et paramédicaux, examens complémentaires<br>Médicaments et équipements médicaux	Coût des transports médicaux utilisés pour transporter le patient vers une structure de soins (p. ex. hôpital, laboratoire d'analyse) et son retour

Les consommations de soins relatives à la prise en charge des patients seront recueillies sur une période de 1 mois auprès de la Caisse Nationale d'Assurance maladie (CNAM). Ces données seront relevées de manière rétrospective et selon une approche bottom-up. Le Numéro d'Inscription au Répertoire (NIR) habituellement utilisé par les organismes d'assurance maladie pour identifier les patients servira de clé d'identification des patients, afin de permettre un appariement direct aux données du Système National des Données de Santé (SNDS). L'utilisation du NIR pour un chainage direct aux données du SNDS est autorisée depuis la parution au journal officiel du 29 mars 2017. De plus, le CHU de Toulouse a obtenu plusieurs avis CNIL favorables pour l'utilisation du NIR en tant que clé d'identification dans des projets de recherche (Décret du JORF n°0075 texte n° 133 en date du 29 mars 2017).
Le NIR sera recueilli par les ARCs dans un fichier contenant un algorithme permettant de crypter instantanément le NIR. Ce fichier, contenant l'ID-Accrochage et le nir, la ddn complète et le sexe sera ensuite envoyé au tiers de confiance qui le transmettra par la suite à la CNAM au moyen de la procédure SAFE. La CNAM renverra par la suite, à l'Unité d'Evaluation médico-Economique du du CHU de Toulouse une calculette ainsi qu'un code d'accès au portail du SNDS. L'UEME sera en charge de générer un numéro d'enquête et de conserver une table de correspondance entre le numéro d'enquête et l'identifiant d'accrochage afin d'analyser les données sur le portail du SNDS, exclusivement ua moyen du numéro d'enquête. L'exploitation de ces données anonymisées sera réalisée par l'unité d'évaluation médico-économique du CHU Toulouse.

8.2.2. VALORISATION DE LA CONSOMMATION DE SOINS

8.2.2.1. COUTS DIRECTS MEDICAUX

Les coûts des séjours hospitaliers seront valorisés à partir du cadre défini par la Tarification à l'Activité (T2A). Les séjours hospitaliers liés à la prise en charge des patients seront valorisés à partir des Groupes Homogènes de Séjours (GHS) correspondant aux Groupes Homogènes de Malades (GHM) complétés des suppléments applicables. Ainsi, les séjours hospitaliers correspondant à la prise en charge d'un patient seront valorisés à partir des GHS et des suppléments correspondants pour les séjours effectués dans le secteur Médecine Chirurgie Obstétrique (MCO).

Pour les séjours effectués en soins de suite et de réadaptation (SSR), la valorisation sera réalisée à l'aide des prix de journée.

Les coûts des soins ambulatoires réalisés en médecine de ville ou dans un établissement de soins du secteur public ou privé seront identifiés et valorisés sur la base de la tarification appliquée par l'assurance maladie. Les tarifs de la Nomenclature Générale des Actes Professionnels (NGAP), de la Classification Commune des Actes Médicaux (CCAM) et de la Nomenclature des Actes de Biologie Médicale (NABM) seront appliqués. Les coûts des traitements médicamenteux et des équipements médicaux seront valorisés sur la base des tarifs de remboursement de l'assurance maladie et séparés entre le montant remboursé par l'assurance maladie et le reste à charge pour le patient.

8.2.2.2. COUTS DIRECTS NON MEDICAUX

Les coûts des transports seront valorisés en tenant compte de la distance existant entre le lieu de résidence du patient et son lieu de prise en charge, du mode de transport utilisé et du niveau de prise en charge. Le tarif reconnu par l'assurance maladie selon le mode de transport utilisé (ambulance, véhicule sanitaire léger, taxi, train ou véhicule personnel) sera appliqué.

Pour les coûts directs médicaux et non médicaux, nous appliquerons le taux de remboursement de l'Assurance Maladie auxquels seront ajoutées ou soustraites les éventuelles franchises et majorations. Puis nous évaluerons également le reste à charge pour les patients.

8.2.2.3. COUT DE PRODUCTION DE LA MISE EN ŒUVRE DE L'ECHOGRAPHIE PULMONAIRE ET CARDIAQUE

L'évaluation du coût de production en lien avec l'utilisation de l'échographie cardiaque et pulmonaire sera réalisée du point de vue des établissements hospitaliers. Nous recueillerons le temps d'utilisation du matériel que nous valoriserons en fonction de l'amortissement. Le temps passé par le personnel à prendre en charge le patient au cours de cet examen sera valorisé au moyen du salaire horaire brut chargé.

8.3. ANALYSES D'EFFICIENCE

L'analyse coût-utilité nous permettra de calculer le ratio coût-utilité différentiel de l'utilisation de l'échographie pulmonaire et cardiaque pour le diagnostic de la dyspnée chez le sujet âgé par rapport à la prise en charge conventionnelle du point de vue de l'assurance maladie. Elle permettra de comparer les conséquences médicales, mesurées en termes de QALY à 1 mois, et des conséquences économiques en termes de soins et bien médicaux (25) (HAS 2020).

L'analyse coût-efficacité nous permettra de calculer le ratio coût-efficacité différentiel de l'utilisation de l'échographie pulmonaire et cardiaque pour le diagnostic de la dyspnée chez le sujet

âgé par rapport à la prise en charge conventionnelle du point de vue de l'assurance maladie. Elle permettra de comparer les conséquences médicales, mesurées en termes de nombre de patients ayant bénéficié d'une thérapie adaptée de manière précoce, et des conséquences économiques en termes de soins et bien médicaux (25).

8.4. ANALYSE D'IMPACT BUDGETAIRE

L'analyse d'impact budgétaire (AIB) est une approche financière destinée à estimer, sur un horizon temporel à court ou moyen terme, les conséquences financières annuelles de l'adoption d'une intervention de santé. Cette estimation résulte du calcul de la différence entre les prévisions de dépenses des scénarios intégrant ou non l'intervention de santé étudiée (HAS 2016) .

L'AIB sera menée sur un horizon temporel de 5 ans, du point de vue de l'assurance maladie et des établissements hospitaliers. Les conséquences financières des différents scénarii seront évaluées annuellement et de manière globale sur les 5 ans.

La population d'intérêt sur laquelle sera menée l'AIB sera les populations cibles et rejointes attendues. Les populations seront décrites pour chaque année en tenant compte des évolutions possibles de leurs estimations. Les populations cibles et rejointes seront estimés par des avis d'experts, majoritairement des centres inclus dans l'étude. Une revue de littérature sera menée pour synthétiser les évolutions d'incidence et de prévalence du cancer du sein en France et au niveau international.

Le calcul de l'impact budgétaire résultera de la comparaison de deux scénarios : un scénario n'intégrant pas l'utilisation de l'échographie pulmonaire et cardiaque sera comparé au scénario l'intégrant. L'intervention substituable à prendre en compte est la prise en charge conventionnelle. La répartition de l'utilisation actuelle des deux types de prise en charge diagnostiques sera étudiée au moyen de la littérature nationale, internationale. L'estimation des évolutions à venir dans la pratique courante, avec et sans l'introduction de l'échographie pulmonaire et cardiaque, à travers de nouvelles répartitions de la population rejointe sera réalisée. Le modèle utilisé pour conduire l'AIB sera un arbre décisionnel.

Le périmètre des coûts ainsi que leur valorisation reposeront sur la même méthode que celle décrite aux chapitres précédents. Comme recommandé par l'HAS, l'actualisation des coûts ne sera pas réalisée.

9. DEROULEMENT DE LA RECHERCHE

9.1. CALENDRIER DE LA RECHERCHE

Durée de la période d'inclusion : 24 mois

Durée de participation de chaque participant : 1 mois

Durée totale de la recherche : 25 mois

9.2. TABLEAU RECAPITULATIF DU SUIVI PARTICIPANT

	Inclusion<br>T 0	Visite H1<br>T 60 min	Visite sortie SU<br>T H4	Appel M1<br>T J30
Inclusion par la procédure d'urgence ou par le consentement éclairé du proche	✓(R)			
Consentement éclairé de poursuite (patient ou proche)		✓(R)	✓(R)	✓(R)
Examen clinique	✓(S)		✓(S)	
Examen échographique (bras intervention)	✓(R)			
Bilan biologique	✓(S)		✓(S)	
Examens para cliniques	✓(S)		✓(S)	
Thérapeutiques engagées		✓(R)	✓(R)	
Recherche des EvI	✓(R)	✓(R)	✓(R)	✓(R)
Entretien téléphonique				✓(R)

R : examen réalisé dans le cadre de la recherche
S : examen réalisé dans le cadre du soin courant

9.3. VISITE D'INCLUSION

9.3.1. RECUEIL DU CONSENTEMENT

Lors de l'admission du patient aux urgences et au vu de son état de santé initial, il sera hors d'état d'exprimer son consentement. Comme la situation d'urgence ne permettra pas de recueillir le consentement du patient, celui d'un membre de sa famille ou celui de la personne de confiance sera recherché s'ils sont présents. Dans le cas contraire, l'inclusion sera réalisée selon la procédure d'urgence conformément à la loi (Art. L.1122-1-3 du Code de la Santé Publique). L'investigateur tracera cette inclusion en urgence sur le formulaire prévu à cet effet.

L'information sera délivrée, dès que possible, à la personne de confiance au sens de l'article L. 1111-6 du code de la santé publique ou, à défaut à la famille ou aux proches. Dès lors que leur état de santé le permettra, les patients concernés seront informés de l'étude et de leurs droits. et leur

consentement leur sera demandé pour la poursuite éventuelle de cette recherche, après un délai de réfléxion d'une heure minimum.

Si aucun consentement n'a été recueilli avant la fin du suivi du patient dans le service d'urgence, l'information se fera auprès du patient dès que son état de santé le permettra et que l'équipe en aura connaissance (ou auprès du proche si le patient n'est toujours pas en état de consentir) par appel téléphonique et transmission de la notice d'information par courrier.

Lors d'un premier appel, l'étude sera alors présentée au patient (ou proche si l'état du patient ne lui permet toujours pas de consentir de manière éclairée) par un investigateur. S'ils acceptent, la Notice d'Information leur sera envoyée par courrier.

Lors d'un second appel, après un délai de réflexion d'au minimum 24 heures, ils pourront alors consentir à la poursuite de l'étude et à l'utilisation des données ou bien exprimer leur refus de consentement le cas échéant. Le consentement oral sera recueilli via un formulaire spécifique qui sera incrémenté dans le dossier médical du patient.

Au vu de la population cible (personne de plus de 65 ans) et de la possibilité d'hospitalisation dans des établissements périphériques après le passage aux urgences, la mise en place d'une procédure de recueil de consentement libre, éclairé et exprès de manière orale a semblé plus appropriée à la situation.

En cas de refus de poursuivre exprimé par un proche ou par le patient lui-même, les données recueillies dans le cadre de la recherche seront effacées.

Dans l'hypothèse où le patient ne serait toujours pas en capacité de donner un consentement libre et éclairé à J30, le consentement du proche (ou personne de confiance) qui aura été recueilli dès que possible sera suffisant pour pouvoir utiliser les données de la recherche.

Lorsqu'un sujet décède et qu'aucun consentement de participation n'a pu être recueilli, les données de ce patient sont perdues et ne peuvent pas être utilisées dans la recherche.

9.3.2. DEROULEMENT DE LA VISITE

La visite d'inclusion est assurée par le médecin investigateur. Elle se tient lors de l'admission du malade en SU, ouverts 24/24h et 7/7J. Avant tout examen lié à la recherche, l'investigateur vérifie les critères d'éligibilité et procède à l'inclusion du patient par la procédure d'urgence.

Après interrogatoire et examen clinique du malade, l'investigateur réalise :

- Le recueil des données d'identification patient
- Le recueil des données démographiques et cliniques: âge, sexe, antécédents cardiaques, respiratoires, diabète, insuffisance rénale, traitement habituel, paramètres d'entrée (tension artérielle, fréquences cardiaques et respiratoires, SpO2, température) auscultation pulmonaire et

cardiaque, présence d'œdème des membres inférieurs, présence de turgescence jugulaire, résultats de l'ECG.

- Le recueil du numéro de sécurité sociale pour l'analyse médico-économique. Celui-ci sera anonymisé par la procédure détaillée en 9.2.1.
- La prescription des examens complémentaires selon le protocole de service
- La prescription des thérapeutiques médicamenteuses urgentes (et recueil de ces dernières) soit non guidée (stratégie standard) soit guidée par l'échographie (stratégie échographie clinique)
- Le choix d'initier un support respiratoire (ventilation non invasive ou oxygénothérapie haut débit) laissé à l'appréciation du médecin selon les protocoles de service.
- Le recueil de l'hypothèse diagnostique initiale (avant rendu des examens complémentaires)

Dans le bras intervention, l'expérience en échographie de l'investigateur ayant réalisé la procédure d'échographie sera recueillie.

9.4. VISITES DE SORTIE DES URGENCES

A la sortie du SU, l'investigateur recueillera les thérapeutiques entreprises lors du passage en SU, les résultats des examens complémentaires et l'orientation post-SU du patient, ainsi que la durée de prise en charge.

Les éléments du dossier d'expertise comprenant les données recueillies lors du passage aux urgences seront saisis dans l'eCRF. Les pièces complémentaires ne pouvant être saisies dans l'eCRF telles que les clichés de radiographie thoracique réalisés dans le cadre du soin seront anonymisés pour être transmis aux experts via une messagerie sécurisée. L'ensemble de ces éléments leur permettra d'effectuer l'analyse centralisée des dossiers afin d'établir le classement diagnostique et l'adéquation/inadéquation thérapeutique pour répondre au critère de jugement principal (tel que décrit dans le paragraphe 4.1).

9.5. VISITE DE FIN DE LA RECHERCHE

La dernière visite de la recherche consiste en un entretien téléphonique d'une durée de 30 minutes environ. Le patient (ou son entourage ou son médecin généraliste) sera contacté à J30 pour évaluer la mortalité à J30 et le nombre de jours vivants hors de l'hôpital à 30 jours. La qualité de vie sera également évaluée au moyen de l'échelle EQ-5D-5L lors de cette visite téléphonique.

Enfin, un questionnaire médico-économique (Annexe 4) portant sur le niveau de diplôme, la catégorie-socio-professionnelle du patient et le niveau de revenu net du patient ou du foyer sera soumis au patient ou à son proche afin de répondre aux critères médico-économiques.

Le nombre de jour d'hospitalisation durant le mois suivant l'inclusion sera recueilli directement au moyen du SNDS.

9.6. REGLES D'ARRET DE LA PARTICIPATION D'UNE PERSONNE A LA RECHERCHE

Un participant peut arrêter sa participation à la recherche à tout moment sans aucune conséquence pour lui ou pour sa prise en charge ultérieure et il peut retirer son consentement à participer à la recherche quel que soit le moment, par contact à l'investigateur local.

A chaque visite, l'investigateur s'assurera que le malade est toujours d'accord pour poursuivre sa participation à l'étude.

Si un patient est exclu de l'étude, qu'il décide de se retirer de l'étude ou qu'il ne consent pas à la poursuite de l'étude et à l'utilisation de ses données après inclusion par la procédure d'urgence, il ne sera pas remplacé.

9.7. CONTRAINTES LIEES A LA RECHERCHE ET INDEMNISATION EVENTUELLE DES PARTICIPANTS

Le patient peut se prêter à une autre recherche simultanément seulement si cette dernière ne modifie pas la prise en charge diagnostique ou thérapeutique dans la première heure de prise en charge au service des urgences.

Il n'y a pas de période d'exclusion.

Aucune indemnisation n'est prévue et le participant ne sera pas inscrit dans le fichier national des personnes qui se prêtent à des recherches.

10. GESTION DES ÉVÉNEMENTS INDÉSIRABLES / EFFETS INDESIRABLES / INCIDENTS

Ce protocole correspond à une recherche impliquant la personne humaine « à risques et contraintes minimes » (catégorie 2 selon l'article L1121 du CSP) qui n'oblige plus les investigateurs à déclarer les événements indésirables « graves » au promoteur.

Les procédures de l'étude (échographie) sont non invasives et dénuées de risques graves.

Les évènements indésirables / effets indésirables / incidents seront à déclarer aux différents circuits de vigilances sanitaires applicables à chaque produit ou pratique concernée (vigilance du soin, pharmacovigilance, matériovigilance, hémovigilance, …) en conformité avec la règlementation en vigueur.

Les déclarants doivent spécifier que le participant est inclus dans un essai clinique et identifier précisément l'essai clinique concerné.

En cas de survenue éventuelle d'événements indésirables / incidents impactant significativement la sécurité des participants et/ou le rapport bénéfices/risques et/ou le bon déroulement de l'étude, le centre investigateur avertira immédiatement le promoteur (CHU de Toulouse) aux coordonnées mentionnées ci-dessous.

La vigilance du promoteur reste joignable pour toute question relative à la sécurité des patients inclus aux coordonnées suivantes :

Nom du responsable de la vigilance pour le promoteur: XXXXXX

CHU de TOULOUSE

11. ASPECTS STATISTIQUES

11.1. CALCUL DE LA TAILLE D'ETUDE

Notre hypothèse envisage une réduction de l'inadéquation de la prise en charge thérapeutique (critère de jugement principal) de 35% à 17,5% (1,16). Dans le cadre d'un essai en stepped-wedge, le nombre de sujets nécessaires a été obtenu en appliquant la méthode de calcul décrite par Hemming K *et al.* (26) en se basant sur les paramètres suivants :

- Le nombre de pas (nombre de temps de mesure) est t + 1 = 8

- Le nombre de cluster randomisé par pas est g = 1

- Le nombre total de clusters est k = g × t = 7

- Le nombre de sujets nécessaire pour rechercher une différence de 35% versus 17,5% avec une puissance de 80% et un risque alpha de 5% bilatéral, dans un essai contrôlé randomisé classique en bras parallèles est de N=196 ,

- Le coefficient de corrélation intra-classe caractérisant la corrélation intra-cluster est de ρ = 0,01. Ce coefficient de corrélation intra-classe a été déterminé par analogie avec des études menées dans un contexte similaire (Service d'urgence) et évaluant d'autres procédures diagnostiques pour des pathologies cardio-pulmonaires (27).

En se basant sur ces paramètres, chaque centre devra recruter 8 patients tous les 3 mois (à chaque pas) pour rechercher une différence de 17,5% entre les deux stratégies d'intervention, avec une puissance de 80% et un risque alpha de 5%. Afin de pallier aux données manquantes et pour anticiper sur une potentiel augmentation du coefficient de corrélation intra-classe nous recruterons un patient en plus par pas par centre (soit 9 patients tous les trois mois). Dans ces conditions l'augmentation du coefficient de corrélation intra-classe n'affectera pas la puissance de l'étude si il reste inférieur à 0.062.

Cela correspond au total à un effectif de 504 patients.

11.2. METHODES STATISTIQUES EMPLOYEES

La cohérence de la base de données sera vérifiée par des contrôles logiques, les erreurs repérées seront corrigées dans la mesure du possible.

Les données manquantes seront décrites de manière détaillée. Les données manquantes concernant les critères de jugement binaires seront traitées en analyses de sensibilité (« worst-case scenario »). Une procédure d'imputation multiple sera envisagée si plus de 10% des patients présentent des données manquantes sur les facteurs de confusion utilisés lors de l'analyse multivariée.

Un flow-chart décrira les inclusions, non-inclusion, sorties d'études, exclusion en cours d'étude.

L'analyse sera réalisée en intention de traiter. Les caractéristiques initiales des centres et des patients seront décrites dans l'ensemble de la population, dans chaque bras de pris en charge, et en fonction des 8 périodes correspondant à chaque « pas » de l'étude. Les variables quantitatives seront décrites selon leurs effectifs, moyenne, écarts-types et distribution en percentile (minimum, maximum, médiane, quartiles). Les variables qualitatives seront décrites en fonction des effectifs et pourcentages des modalités de réponse. Comme il s'agit d'une randomisation par cluster, la distribution des facteurs de confusion potentiels entre les deux stratégies comparées n'est pas garantie par la randomisation. La distribution des caractéristiques initiales seront donc comparés entre les deux stratégies, en prenant en compte la corrélation intra-cluster à l'aide de modèles à effets mixtes. L'utilisation de l'échographie dans le groupe « prise en charge standard » sera décrite dans l'ensemble, et en fonction des 8 périodes correspondant à chaque « pas » de l'étude. Le coefficient de corrélation intra-classe sera calculé. S'il est supérieur à 0,062 une analyse post-hoc de la puissance de l'étude sera effectuée.

Pour l'analyse du critère de jugement principal, un modèle à effet mixte sera appliqué pour comparer l'inadéquation thérapeutique initiale entre les deux stratégies comparées, à l'aide d'une régression logistique à effet mixte incluant l'effet centre comme intercept aléatoire. Ce modèle sera ajusté sur la période de recrutement (prise en compte comme facteur à effet fixe). Une interaction centre*temps sera recherchée en testant un effet « pente aléatoire » du délai entre le début de l'étude et la date de recrutement. Le modèle sera ajusté sur les facteurs de confusion potentiels, notamment âge, sexe, antécédents (cardiopathie, respiratoire, rénal, diabète démence) et diagnostic final.

La même stratégie d'analyse sera appliquée pour les critères de jugement secondaires, en utilisant une régression logistique à effets mixte pour les critères de jugement binaires (« présence d'une erreur au diagnostic initial », « mortalité à J30 ») , un modèle linéaire à effet mixte pour les critères

quantitatives continus (« durée de passage aux urgences », « durée d'hospitalisation »), ou un modèle de Poisson à effets mixtes pour les variables de compte (« nombre de jours en vie hors de l'hôpital »). Si besoin, une transformation logarithmique sera appliquée aux variables « durée de passage aux urgences » et « durée d'hospitalisation » afin de normaliser la distribution.

11.3. METHODES STATISTIQUES EMPLOYEES POUR LA PARTIE ECONOMIQUE

Les coûts globaux en soins des patients seront décrits dans les deux bras. Pour ces analyses descriptives, les variables quantitatives seront décrites par les moyennes, écarts-types, minimum, maximum, quartiles et médiane. Une comparaison des coûts de prise en charge entre les deux bras sera réalisée. Elle reposera sur l'utilisation de tests statistiques en série indépendante (test de T Student ou test de Mann-Whitney). Nous testerons également l'effet de certaines variables d'intérêts sur les coûts de prise en charge, sur l'efficacité et sur les ratios d'efficience. Nous testerons particulièrement l'effet des disparités socio-économique et territoriale sur les coûts de prise en charge et sur les ratios d'efficience. Pour étudier les disparités socio-économique, nous prendrons en considération le niveau de revenu, le niveau d'éducation et la catégorie socio-professionnelle (avant la retraite) su patient. Nous utiliserons des modèles mixtes pour étudier l'impact des variables d'intérêts sur le coût de prise en charge et des méthodes d'impact net de l'équité pour estimer l'impact de ces caractéristiques sur le ratio d'efficience.

Des analyses de sensibilité déterministe et probabiliste seront réalisées. Dans le cadre de l'analyse de sensibilité déterministe nous étudierons la robustesse des résultats en mesurant l'impact sur le résultat final de la variation de différents paramètres de coûts et d'efficacité (25). L'analyse de sensibilité probabiliste, sera réalisée à l'aide de la méthode du bootstrap non-paramétrique. Elle nous permettra d'identifier l'incertitude autour de l'ICER en estimant son intervalle de confiance (28). De plus, une courbe d'acceptabilité sera construite. Elle permet d'apprécier, de manière visuelle, la relation entre l'incertitude du ratio coût-utilité différentiel et la valeur seuil consentit par le payeur (i.e. Disposition à Payer Collective). Elle définit la probabilité qu'une stratégie soit efficiente, en fonction de la valeur seuil définit par la collectivité (29).

L'impact budgétaire sera modélisé en utilisant l'approche des cohortes fermées et reposera sur un modèle de type arbre décisionnel. Les résultats de l'AIB seront présentés dans un tableau, année par année et au total sur les 5 ans, en termes de coûts par scénario et de différence de coûts entre les scénarii. Des analyses de sensibilité déterministes seront réalisées. Des analyses en scénario seront réalisées sur les paramètres les plus importants de l'AIB (c.-à-d. taille de la population cible, les parts de marché et le prix revendiqué de l'intervention étudiée, taux de diffusion de la technique).

12. GESTION ET TRAITEMENT DES DONNEES ET DOCUMENTS SOURCE

12.1. ACCES AUX DONNEES

L'acceptation de la participation au protocole implique que les investigateurs mettront à disposition les documents et données individuelles strictement nécessaires au suivi, au contrôle de qualité et à l'audit de la recherche, à la disposition des personnes ayant un accès à ces documents conformément aux dispositions législatives et réglementaires en vigueur.

12.2. DONNEES SOURCE

Ensemble des informations figurant dans des documents originaux, ou dans des copies authentifiées de ces documents, relatif aux examens cliniques, aux observations ou à d'autres activités menées dans le cadre d'une recherche et nécessaires à la reconstitution et à l'évaluation de la recherche. Les documents dans lesquels les données sources sont enregistrées sont appelés les documents sources.

12.2.1. DONNEES ISSUES DU SNDS

Les données de l'étude feront l'objet d'un rapprochement avec les données issues du SNDS par l'utilisation du numéro d'inscription au répertoire national d'identification des personnes physiques (NIR), du sexe et de la date de naissance complète des participants.
Le circuit d'appariement sera conforme à la fiche pratique « multi-centres / eCRF sans NIR » publiée par la CNIL. A cet égard, les données identifiantes seront transmises, via un tiers de confiance qui a pour rôle de centraliser les données nécessaires à l'appariement, à la CNAM qui effectuera le rapprochement avec les données du SNDS.
Ces données seront chiffrées au sein des centres et transmises au tiers sous forme de fichiers chiffrés. Les algorithmes et les procédures de gestion de clés seront conformes à l'annexe B1 du référentiel général de sécurité.
Après accord du patient, conformément au décret no 2018-173 du 9 mars 2018 autorisant la création d'un traitement de données à caractère personnel relatif à l'activité et à la consommation de soins dans les établissements ou services médico-sociaux, le numéro de Sécurité Sociale (NIR : Numéro d'Inscription au Répertoire) du patient sera recueilli sur un formulaire indépendant et saisi à part des autres données du CRF sur une base contenant aussi l'identifiant d'accrochage généré par l'e-CRF, la date de naissance complète et le genre du patient. Cette base sera ensuite envoyée de manière sécurisée au tiers de confiance (ORU-Occitanie) qui transmettra les données à la CNAM. L'UEME sera en charge de générer un numéro d'enquête et de conserver une table de correspondance entre le numéro d'enquête et l'identifiant d'accrochage afin d'analyser les données sur le portail du SNDS avec le numéro d'enquête exclusivement. Le promoteur s'engage à demander l'accord de toutes les autorités compétentes nécessaires pour avoir accès au SNDS, soit le CPP (Comité de Protection des Personnes), et la CNIL (Commission Nationale de l'Informatique et des Libertés).

12.3. CONFIDENTIALITE DES DONNEES

Conformément aux dispositions législatives en vigueur, les personnes ayant un accès direct aux données source prendront toutes les précautions nécessaires en vue d'assurer la confidentialité des informations relatives aux médicaments expérimentaux, aux recherches, aux personnes qui s'y prêtent et notamment en ce qui concerne leur identité ainsi qu'aux résultats obtenus. Ces personnes, au même titre que les investigateurs eux-mêmes, sont soumises au secret professionnel.

Pendant la recherche ou à son issue, les données recueillies sur les personnes qui s'y prêtent et transmises au promoteur par les investigateurs (ou tous autres intervenants spécialisés) seront rendues anonymes. Elles ne doivent en aucun cas faire apparaître en clair les noms des personnes concernées ni leur adresse.

Seule la première lettre du nom et du prénom du sujet seront enregistrées, accompagnées d'un numéro des centres et un numéro codé propre à la recherche indiquant l'ordre d'inclusion des sujets.

Le promoteur s'assurera que chaque personne qui se prête à la recherche a donné son accord par écrit pour l'accès aux données individuelles la concernant et strictement nécessaires au contrôle de qualité de la recherche.

La figure 1 schématise le circuit des données entre le centre investigateur, le tiers de confiance, la CNAM et l'UEME du CHU de Toulouse. Ce circuit correspond au Circuit validé par la CNIL dans son guide pratique édité en juin 2024 (30).

Figure 1. Circuit du NIR

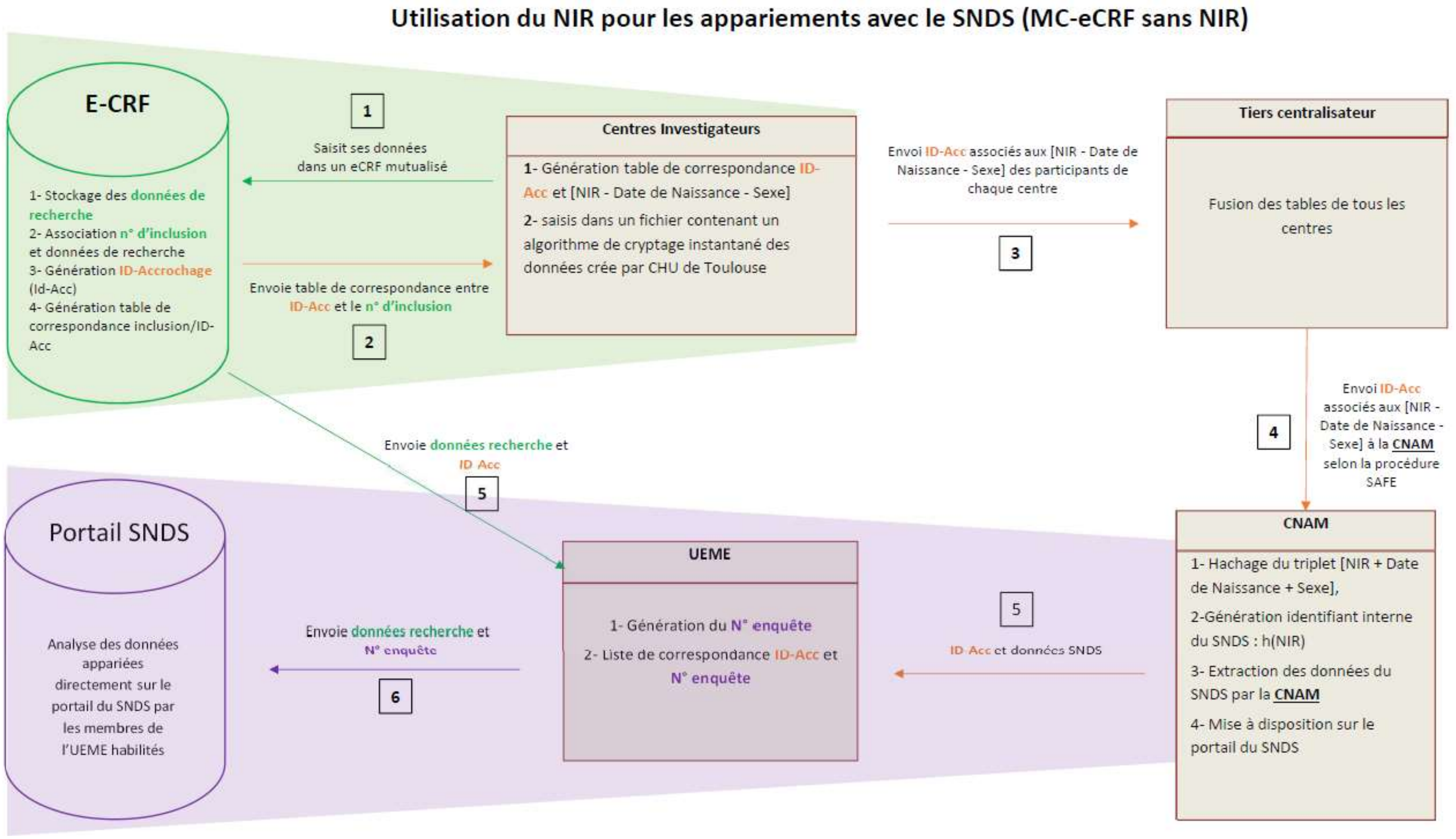

13. CONTROLE ET ASSURANCE QUALITE

13.1. CONSIGNES POUR LE RECUEIL DES DONNEES

Toutes les informations requises par le protocole doivent être consignées sur les cahiers d'observation et une explication doit être apportée pour chaque donnée manquante. Les données doivent être recueillies au fur et à mesure qu'elles sont obtenues, et transcrites dans ces cahiers de façon nette et lisible.
Les données seront recueillies sur un CRF électronique conçu par le coordinateur de l'étude en collaboration avec l'USMR. Les données de la base seront complétées par les médecins avec l'aide d'un technicien de recherche clinique local.

13.2. CONTROLE QUALITE

Un attaché de recherche clinique mandaté par le promoteur visite de façon régulière chaque centre investigateur, lors de la mise en place de la recherche, une ou plusieurs fois en cours de recherche selon le rythme des inclusions et en fin de recherche. Lors de ces visites, et conformément au plan de monitorage basé sur le risque (participant, logistique, impact, ressources), les éléments suivants seront revus :

- consentement éclairé,
- respect du protocole de la recherche et des procédures qui y sont définies,
- qualité des données recueillies dans le cahier d'observation : exactitude, données manquantes, cohérence des données avec les documents source (dossiers médicaux, carnets de rendez-vous, originaux des résultats de laboratoire, etc,…),
- gestion des produits éventuels.

Toute visite fera l'objet d'un rapport de monitorage par compte-rendu écrit.

13.3. GESTION DES DONNEES

Les données seront saisies dans les cahiers d'observation informatisé (e-CRF) par les investigateurs et les techniciens de recherche clinique. Ce CRF sera mis en place à l'aide du logiciel Clinsight par l'équipe de data-management de l'USMR, qui gèrera les modalités de codage des données, le plan de validation des données, le gel de base, le transfert et l'archivage des données.
Le fichier de la recherche sera conservé durant toute la période de la recherche et bien entendu la période réglementaire liée aux données dans le cadre d'une recherche.

13.4. AUDIT ET INSPECTION

Un audit peut être réalisé à tout moment par des personnes mandatées par le promoteur et indépendantes des personnes menant la recherche. Il a pour objectif de vérifier la sécurité des participants et le respect de leurs droits, le respect de la réglementation applicable et la fiabilité des données s

Une inspection peut également être diligentée par une autorité compétente (ANSM pour la France ou EMA dans le cadre d'un essai européen par exemple).

L'audit, aussi bien que l'inspection, pourront s'appliquer à tous les stades de la recherche, du développement du protocole à la publication des résultats et au classement des données utilisées ou produites dans le cadre de la recherche.

Les investigateurs acceptent de se conformer aux exigences du promoteur en ce qui concerne un audit et à l'autorité compétente pour une inspection de la recherche.

14. CONSIDERATIONS ETHIQUES ET REGLEMENTAIRES

Le promoteur et l'(es) investigateur(s) s'engagent à ce que cette recherche soit réalisée en conformité avec la loi n°2012-300 du 5 mars 2012 relative aux recherches impliquant la personne humaine, ainsi qu'en accord avec les Bonnes Pratiques Cliniques (I.C.H. version 4 du 9 novembre 2016 et décision du 24 novembre 2006) et la déclaration d'Helsinki (qui peut être retrouvée dans sa version intégrale sur le site http://www.wma.net).

La recherche est conduite conformément au présent protocole. Hormis dans les situations d'urgence nécessitant la mise en place d'actes thérapeutiques précis, l'(es) investigateur(s) s'engage(nt) à respecter le protocole en tous points en particulier en ce qui concerne le recueil du consentement et la notification et le suivi des événements indésirables graves.

Cette recherche a reçu l'avis favorable du Comité de Protection des Personnes (CPP) Est IV

Le CHU de Toulouse, promoteur de cette recherche, a souscrit un contrat d'assurance en responsabilité civile auprès de la société Lloyds Insurance Company S.A. (conformément aux dispositions du code de la santé publique).

Les données enregistrées à l'occasion de cette recherche font l'objet d'un traitement informatisé au CHU de Toulouse dans le respect de la loi n°78-17 du 6 janvier 1978 relative à l'informatique, aux fichiers et aux libertés modifiée par la loi n° 2018-493 du 20 juin 2018 relative à la protection des données personnelles et au règlement général sur la protection des données (règlement UE 2016/679).

Cette recherche ne rentre pas dans le cadre de la « Méthodologie de référence » (MR-001) en raison de l'utilisation des données issues du SNDS. Le CHU de Toulouse, responsable du traitement des données, demandera une autorisation de recherche à la Commission Nationale de l'Informatique et des Libertés (CNIL).

- Cette recherche est enregistrée sur le site http://clinicaltrials.gov/

MODIFICATIONS AU PROTOCOLE

Toute modification substantielle, c'est à dire toute modification de nature à avoir un impact significatif sur la protection des personnes, sur les conditions de validité et sur les résultats de la recherche, sur la qualité et la sécurité des produits expérimentés, sur l'interprétation des documents scientifiques qui viennent appuyer le déroulement de la recherche ou sur les modalités de conduite de celle-ci, fait l'objet d'un amendement écrit qui est soumis au promoteur ; celui-ci doit obtenir, préalablement à sa mise en œuvre, un avis favorable du CPP et, le cas échéant, une autorisation de l'ANSM.

Les modifications non substantielles, c'est à dire celles n'ayant pas d'impact significatif sur quelque aspect de la recherche que ce soit, sont communiquées au CPP à titre d'information.

Toutes les modifications sont validées par le promoteur, et par tous les intervenants de la recherche concernés par la modification, avant soumission au CPP et, le cas échéant, à l'ANSM. Cette validation peut nécessiter la réunion de tout comité constitué pour la recherche.

Toutes les modifications au protocole doivent être portées à la connaissance de tous les investigateurs qui participent à la recherche. Les investigateurs s'engagent à en respecter le contenu.

Toute modification qui modifie la prise en charge des participants ou les bénéfices, risques et contraintes de la recherche fait l'objet d'une nouvelle note d'information et d'un nouveau formulaire de consentement dont le recueil suit la même procédure que celle précitée.

15. CONSERVATION DES DOCUMENTS ET DES DONNEES RELATIFS A LA RECHERCHE

Les documents suivants relatifs à cette recherche sont archivés par l'investigateur conformément aux Bonnes Pratiques Cliniques :

- ***pour une durée de 15 ans suivant la fin de la recherche*** (recherches portant sur des médicaments, des dispositifs médicaux ou des dispositifs médicaux de diagnostic in vitro ou recherches ne portant pas sur un produit mentionné à l'article L.5311-1 du code de la santé publique),

  - Le protocole et les modifications éventuelles au protocole
  - Les cahiers d'observation (copies)
  - Les dossiers source des participants ayant signé un consentement

- Tous les autres documents et courriers relatifs à la recherche
- L'exemplaire original des consentements éclairés signés des participants

Tous ces documents sont sous la responsabilité de l'investigateur pendant la durée réglementaire d'archivage.

Aucun déplacement ou destruction ne pourra être effectué sans l'accord du promoteur. Au terme de la durée réglementaire d'archivage, le promoteur sera consulté pour destruction. Toutes les données, tous les documents et rapports pourront faire l'objet d'audit ou d'inspection.

16. RAPPORT FINAL

Dans un délai d'un an suivant la fin de la recherche ou son interruption, un rapport final sera établi et signé par le promoteur et l'investigateur. Ce rapport sera tenu à la disposition de l'autorité compétente. Le promoteur transmettra au CPP et, le cas échéant, à l'ANSM les résultats de la recherche sous forme d'un résumé du rapport final dans un délai d'un an après la fin de la recherche.

17. REGLES RELATIVES A LA PUBLICATION

L'analyse des données fournies par les centres investigateurs est réalisée par USMR du CHU de Toulouse. Cette analyse donne lieu à un rapport écrit qui est soumis au promoteur, qui transmettra au Comité de Protection des Personnes et à l'autorité compétente.

Toute communication écrite ou orale des résultats de la recherche doit recevoir l'accord préalable de l'investigateur coordonnateur et, le cas échéant, de tout comité constitué pour la recherche.
L'investigateur coordonnateur/principal s'engage à mettre à disposition du public les résultats de la recherche aussi bien négatifs et non concluants que positifs.

La publication des résultats principaux mentionne le nom du promoteur, de tous les investigateurs ayant inclus ou suivi des participants dans la recherche, des méthodologistes, biostatisticiens et data managers ayant participé à la recherche, des vigilants ayant participé à l'analyse de la sécurité des participants, des membres du comité constitué d'experts pour la recherche et la participation de la source de financement. Il sera tenu compte des règles internationales d'écriture et de publication (31).
L'authorship proposé pour l'article clinique sera le suivant et tiendra compte du nombre de patients inclus par les centres classés pour l'occasion par ordre décroissant d'inclusions :
Balen F, Investigateur n°1, Investigateur n°3, Investigateur n°5, Delmas C, Noel-Savina E, Dubucs X, Hebrard M, Costa N, Investigateur n°6, Investigateur n°4, Investigateur n°2, Shourick J.
Cette publication respectera les critères de rapport du CONSORT / Stepped Wedge (32).

L'analyse médico-économique fera l'objet d'un article à part entière, dont l'authorship sera le suivant : Costa N, Delmas C, Dubucs X, Investigateur n°1, Investigateur n°3, Investigateur n°5, Hebrard M, Investigateur n°6, Investigateur n°4, Investigateur n°2, Shourick J, Noel-Savina E, Balen F.

17.1. COMMUNICATION DES RESULTATS AUX PARTICIPANTS

Conformément à la loi n°2002-303 du 4 mars 2002, les participants sont informés, à leur demande, des résultats globaux de la recherche.

17.2. CESSION DES DONNEES

La gestion des données est assurée par le CHU de Toulouse. Les conditions de cession de tout ou partie de la base de données de la recherche sont décidées par le promoteur de la recherche et font l'objet d'un contrat écrit.

REFERENCES BIBLIOGRAPHIQUES

1. Ray P, Birolleau S, Lefort Y, Becquemin M-H, Beigelman C, Isnard R, et al. Acute respiratory failure in the elderly: etiology, emergency diagnosis and prognosis. Crit Care. 2006 May 24;10(3):R82.
2. Nielsen LS, Svanegaard J, Wiggers P, Egeblad H. The yield of a diagnostic hospital dyspnoea clinic for the primary health care section. J Intern Med. 2001 Nov;250(5):422–428.
3. Christiaens H, Charpentier S, Houze-Cerfon C-H, Balen F. Winter virus season impact on acute dyspnoea in the emergency department. Clin Respir J. 2019 Nov;13(11):722–727.
4. Laribi S, Keijzers G, van Meer O, Klim S, Motiejunaite J, Kuan WS, et al. Epidemiology of patients presenting with dyspnea to emergency departments in Europe and the Asia-Pacific region. Eur J Emerg Med. 2019 Oct;26(5):345–349.
5. Ponikowski P, Voors AA, Anker SD, Bueno H, Cleland JGF, Coats AJS, et al. 2016 ESC Guidelines for the diagnosis and treatment of acute and chronic heart failure: The Task Force for the diagnosis and treatment of acute and chronic heart failure of the European Society of Cardiology (ESC)Developed with the special contribution of the Heart Failure Association (HFA) of the ESC. Eur Heart J. 2016 Jul 14;37(27):2129–2200.
6. Metlay JP, Waterer GW, Long AC, Anzueto A, Brozek J, Crothers K, et al. Diagnosis and Treatment of Adults with Community-acquired Pneumonia. An Official Clinical Practice Guideline of the American Thoracic Society and Infectious Diseases Society of America. Am J Respir Crit Care Med. 2019 Oct 1;200(7):e45–e67.
7. Viniol C, Vogelmeier CF. Exacerbations of COPD. Eur Respir Rev. 2018 Mar 31;27(147).
8. Konstantinides SV, Torbicki A, Agnelli G, Danchin N, Fitzmaurice D, Galiè N, et al. 2014 ESC guidelines on the diagnosis and management of acute pulmonary embolism. Eur Heart J. 2014 Nov 14;35(43):3033–69, 3069a.
9. Renier W, Winckelmann KH, Verbakel JY, Aertgeerts B, Buntinx F. Signs and symptoms in adult patients with acute dyspnea: a systematic review and meta-analysis. Eur J Emerg Med. 2018 Feb;25(1):3–11.
10. Matsue Y, Damman K, Voors AA, Kagiyama N, Yamaguchi T, Kuroda S, et al. Time-to-Furosemide Treatment and Mortality in Patients Hospitalized With Acute Heart Failure. J Am Coll Cardiol. 2017 Jun 27;69(25):3042–3051.
11. Seymour CW, Gesten F, Prescott HC, Friedrich ME, Iwashyna TJ, Phillips GS, et al. Time to Treatment and Mortality during Mandated Emergency Care for Sepsis. N Engl J Med. 2017 Jun 8;376(23):2235–2244.
12. Kelly A-M, Keijzers G, Klim S, Craig S, Kuan WS, Holdgate A, et al. Epidemiology and outcome of older patients presenting with dyspnoea to emergency departments. Age Ageing. 2021 Jan 8;50(1):252–257.
13. Mueller C, Scholer A, Laule-Kilian K, Martina B, Schindler C, Buser P, et al. Use of B-type natriuretic peptide in the evaluation and management of acute dyspnea. N Engl J Med. 2004 Feb 12;350(7):647–654.
14. Mueller C, Laule-Kilian K, Frana B, Rodriguez D, Rudez J, Scholer A, et al. The use of B-type natriuretic peptide in the management of elderly patients with acute dyspnoea. J Intern Med. 2005 Jul;258(1):77–85.
15. Staub LJ, Mazzali Biscaro RR, Kaszubowski E, Maurici R. Lung Ultrasound for the Emergency Diagnosis of Pneumonia, Acute Heart Failure, and Exacerbations of Chronic Obstructive Pulmonary Disease/Asthma in Adults: A Systematic Review and Meta-analysis. J Emerg Med. 2019 Jan;56(1):53–69.
16. Russell FM, Ehrman RR, Cosby K, Ansari A, Tseeng S, Christain E, et al. Diagnosing acute heart failure in patients with undifferentiated dyspnea: a lung and cardiac ultrasound (LuCUS) protocol. Acad Emerg Med. 2015 Feb;22(2):182–191.

17. De Carvalho H, Javaudin F, Le Bastard Q, Boureau A-S, Montassier E, Le Conte P. Effect of chest ultrasound on diagnostic workup in elderly patients with acute respiratory failure in the emergency department: a prospective study. Eur J Emerg Med. 2021 Jan 1;28(1):29–33.
18. Balen F, Houze Cerfon C-H, Lauque D, Hebrad M, Legourrierec T, Delmas C, et al. Diagnostic performances of lung ultrasound associated with inferior vena cava assessment for the diagnosis of acute heart failure in elderly emergency patients: a diagnostic study. Eur J Emerg Med. 2020 Nov 11;
19. Martinez M, Duchenne J, Bobbia X, Brunet S, Fournier P, Miroux P, et al. Deuxième niveau de compétence pour l'échographie clinique en médecine d'urgence. Recommandations de la Société française de médecine d'urgence par consensus formalisé. Ann Fr Med Urgence. 2018 Jun;8(3):193–202.
20. Riishede M, Lassen AT, Baatrup G, Pietersen PI, Jacobsen N, Jeschke KN, et al. Point-of-care ultrasound of the heart and lungs in patients with respiratory failure: a pragmatic randomized controlled multicenter trial. Scand J Trauma Resusc Emerg Med. 2021 Apr 26;29(1):60.
21. Zieleskiewicz L, Lopez A, Hraiech S, Baumstarck K, Pastene B, Di Bisceglie M, et al. Bedside POCUS during ward emergencies is associated with improved diagnosis and outcome: an observational, prospective, controlled study. Crit Care. 2021 Jan 22;25(1):34.
22. Vauthier C, Chabannon M, Markarian T, Taillandy Y, Guillemet K, Krebs H, et al. Point-of-care chest ultrasound to diagnose acute heart failure in emergency department patients with acute dyspnea: diagnostic performance of an ultrasound-based algorithm. Emergencias. 2021 Dec;33(6):441–446.
23. Herdman M, Gudex C, Lloyd A, Janssen M, Kind P, Parkin D, et al. Development and preliminary testing of the new five-level version of EQ-5D (EQ-5D-5L). Qual Life Res. 2011 Dec;20(10):1727–1736.
24. Andrade LF, Ludwig K, Goni JMR, Oppe M, de Pouvourville G. A French Value Set for the EQ-5D-5L. Pharmacoeconomics. 2020 Apr;38(4):413–425.
25. Drummond MF, Barbieri M, Wong JB. Analytic choices in economic models of treatments for rheumatoid arthritis: What makes a difference? Med Decis Making. 2005 Oct;25(5):520–533.
26. Hemming K, Taljaard M. Sample size calculations for stepped wedge and cluster randomised trials: a unified approach. J Clin Epidemiol. 2016 Jan;69:137–146.
27. Briggs AH, Wonderling DE, Mooney CZ. Pulling cost-effectiveness analysis up by its bootstraps: a non-parametric approach to confidence interval estimation. Health Econ. 1997 Aug;6(4):327–340.
28. Fenwick E, Byford S. A guide to cost-effectiveness acceptability curves. Br J Psychiatry. 2005 Aug;187(02):106–108.
29. CNIL. Guide pratique. Modalités de circulation du NIR pour la recherche en santé au fins d'appariement de données avec le SNDS. CNIL. Décembre 2020 (https://www.cnil.fr/sites/default/files/atoms/files/guide_pratique_circuits_nir_recherche_en_sante.pdf)
30. https://www.cnil.fr/sites/cnil/files/2024-06/fiche_pratique_circuit_nir_circuit_multi-centres_centre_unique_ecrf_sans_nir.pdf 4.
31. Hemming K, Taljaard M, McKenzie JE, Hooper R, Copas A, Thompson JA, et al. Reporting of stepped wedge cluster randomised trials: extension of the CONSORT 2010 statement with explanation and elaboration. BMJ. 2018 Nov 9;363:k1614.

ANNEXES

ANNEXE 1 : EVALUATION CLINICO-ECHOGRAPHIQUE DU PATIENT AGE EN DETRESSE RESPIRATOIRE

Evaluation initiale à l'admission permet d'initier les thérapeutiques adaptées. A évaluer à l'admission.
(Attention : diagnostics associés dans 40% des cas !)
(cf infra)

Evaluer la participation cardiaque
(2 critères sur 3)
☐ Clinique compatible (Killip ≥2 et OMI bilatéraux et TJ/RHJ)
☐ Profil B échographique
☐ Pression de remplissage élevées :
- E/A > 2 si FEVG altérée ou
- E/e' > 12 quel que soit la FEVG

→
- Furosémide en bolus (40 mg si naïf, 80 mg si non, à répéter toutes les 1h selon évolution clinique et échographique)
- Risordan titration (3 mg toutes les 5 minutes pour TAS ≤ 110 mmHg) puis

↓

Evaluer le risque d'embolie pulmonaire
(1 critère sur 2)
☐ Visualisation de thrombus dans les cavités droites ou signes de cœur pulmonaire aigu
☐ Incompressibilité veineuse aux membres inférieurs

→ Anticoagulation efficace dans l'attente angio-TDM

↓

Evaluer l'infection pulmonaire
(1 critère sur 2)
☐ T° > 37,8°C ou expectoration purulentes
☐ Profil Bb et/ou Profil C

→ Antibiothérapie

↓

Evaluer la participation du bronchospasme
(2 critères sur 3)
☐ ATCD BPCO ou Asthme avéré
☐ Sibilants bilatéraux
☐ Absence de grandes lignes B

→ Aérosols par B2-mimétiques

ANNEXE 2 : DOSSIER EXPERT

EXPERTISE LUC REED

Numéro identification patient : ………….

Diagnostic final :

- OAP ☐
- Décompensation BPCO ☐
- Asthme ☐
- Embolie pulmonaire ☐
- Pneumopathie ☐
- Autre ☐ (Précisez : …………………………………..……..)

Adéquation des thérapeutiques administrées au SU au diagnostic final ?

Oui ☐ Non ☐ NSPP ☐

Données patient

Age : ……… ans Sexe : F ☐ M ☐

Antécédents :

- Cardiopathie : Non ☐ Oui ☐ Si oui, type :
  - Ischémique ☐
  - Valvulaire ☐
  - Rythmique ☐
  - Hypertrophique ☐
  - Dilatée (non classée plus haut) ☐
  - Autre : ……………………… ☐

- Pathologie respiratoire : Non ☐ Oui ☐ Si oui, type :
  - Asthme ☐
  - BPCO ☐
  - Emphysème ☐
  - Cancer pulmonaire ☐
  - Autre ☐

- Insuffisance rénale : Non ☐ Oui ☐
- Diabète : Non ☐ Oui ☐
- Démence : Non ☐ Oui ☐

Traitement habituel :

Nom de la spécialité	Posologie quotidienne	Nom de la spécialité	Posologie quotidienne

Support respiratoire au domicile :
- Oxygène : Non ☐ Oui ☐(débit : .../min ; durée/j : ...h)
- VNI : Non ☐ Oui ☐

Données cliniques

<u>Paramètres d'entrée :</u> TA : ……./……. mmHg FC : ……. bpm T° = ……. °C
FR : …….. mvt/min SpO2= …….% AA
SpO2=…..% ss … l/min

<u>Auscultation pulmonaire :</u>

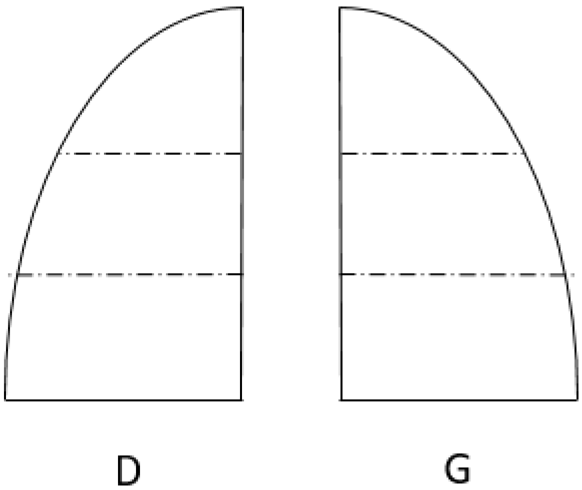

Annotez les 6 champs pulmonaires :

- N : normale
- S : sibilants
- C : crépitants

<u>Souffle auscultatoire :</u> Non ☐ Oui ☐ :

- Mitral ☐ : Systolique ☐ Diastolique ☐
- Aortique ☐ : Systolique ☐ Diastolique ☐

<u>Œdème(s) des membres inférieurs :</u> Absent/minimes ☐
Bilatéraux ☐ : Modérés ☐ Majeurs ☐
Unilatéral ☐

<u>Examen des jugulaires :</u> Pas de turgescence ☐
Reflux Hépato-jugulaire ☐
Turgescence spontanée ☐

<u>ECG à joindre au dossier expert</u>

Données paracliniques

Biologie :

- Hb : ……… g/L - Créatininémie : ……… mmol/L - pH artériel : ……
- GB : ……… G/L - NT-Pro-BNP : ……….. ng/L - pO2 art : ………..
- PNN : …… G/L - CRP : ……………….. - pCO2 art : ………

Radiographie thoracique à joindre au dossier expert

CR TDM thoracique (par radiologue) (si réalisé) :

Thérapeutiques administrées au SU :

ANNEXE 3 : EQ-5D-5L

Pour chaque rubrique, veuillez cocher UNE case, celle qui décrit le mieux votre santé AUJOURD'HUI.

MOBILITÉ

Je n'ai aucun problème pour me déplacer à pied ₁❑
J'ai des problèmes légers pour me déplacer à pied ₂❑
J'ai des problèmes modérés pour me déplacer à pied ₃❑
J'ai des problèmes sévères pour me déplacer à pied ₄❑
Je suis incapable de me déplacer à pied ₅❑

AUTONOMIE DE LA PERSONNE

Je n'ai aucun problème pour me laver ou m'habiller tout(e) seul(e) 1❑
J'ai des problèmes légers pour me laver ou m'habiller tout(e) seul(e) 2❑
J'ai des problèmes modérés pour me laver ou m'habiller tout(e) seul(e) 3❑
J'ai des problèmes sévères pour me laver ou m'habiller tout(e) seul(e) 4❑
Je suis incapable de me laver ou de m'habiller tout(e) seul(e) 5❑

ACTIVITES COURANTES *(p. ex., travail, études, travaux domestiques, activités familiales ou loisirs)*

Je n'ai aucun problème pour accomplir mes activités courantes 1❑
J'ai des problèmes légers pour accomplir mes activités courantes 2❑
J'ai des problèmes modérés pour accomplir mes activités courantes 3❑
J'ai des problèmes sévères pour accomplir mes activités courantes 4❑
Je suis incapable d'accomplir mes activités courantes 5❑

DOULEURS / GÊNE

Je n'ai ni douleur ni gêne 1❑
J'ai des douleurs ou une gêne légère(s) 2❑
J'ai des douleurs ou une gêne modérée(s) 3❑
J'ai des douleurs ou une gêne sévère(s) 4❑
J'ai des douleurs ou une gêne extrême(s) 5❑

ANXIÉTÉ / DÉPRESSION

Je ne suis ni anxieux(se) ni déprimé(e) 1❑
Je suis légèrement anxieux(se) ou déprimé(e) 2❑
Je suis modérément anxieux(se) ou déprimé(e) 3❑
Je suis sévèrement anxieux(se) ou déprimé(e) 4❑
Je suis extrêmement anxieux(se) ou déprimé(e) 5❑

Echelle visuelle analogique (EQ-VAS) :

- Nous aimerions savoir dans quelle mesure votre santé est bonne ou mauvaise AUJOURD'HUI.
- Cette échelle est numérotée de 0 à 100.
- 100 correspond à la <u>meilleure</u> santé que vous puissiez imaginer.
  0 correspond à la <u>pire</u> santé que vous puissiez imaginer.
- Veuillez faire une croix (X) sur l'échelle afin d'indiquer votre état de santé AUJOURD'HUI.
- Maintenant, veuillez noter dans la case ci-dessous le chiffre que vous avez coché sur l'échelle.

VOTRE SANTÉ AUJOURD'HUI = ☐

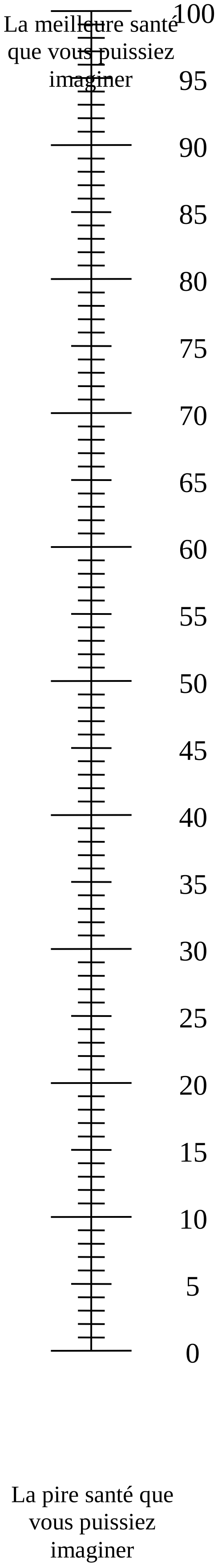

ANNEXE 4: QUESTIONNAIRE MEDICO-ECONOMIQUE

Quel est le plus haut diplôme que le patient a obtenu ?

1- ☐ Aucun diplôme (ou CEP)
2- ☐ Brevet des collèges
3- ☐ CAP, BEP ou équivalent
4- ☐ Baccalauréat, brevet professionnel ou équivalent
5- ☐ Supérieur court (niveau bac + 2)
6- ☐ Supérieur long (supérieur à bac + 2)

A quelle catégorie socio-professionnelle appartient le patient ?

1- ☐ Agriculteurs exploitants
2- ☐ Artisans, commerçants et chefs d'entreprise
3- ☐ Cadres et professions intellectuelles supérieures
4- ☐ Professions Intermédiaires
5- ☐ Employés
6- ☐ Ouvriers
7- ☐ Retraités
8- ☐ Autres personnes sans activité professionnelle

Quel est le revenu net par mois du patient ou du foyer si le patient est en couple?

1- ☐ < 1000 €
2- ☐ 1000€ ≤ x < 2000€
3- ☐ 2000€ ≤ x <3000€
4- ☐ ≥ 3000€
