# Supplementary material for: Lung and cardiac ultrasound for respiratory distress in the elderly: study protocol of the LUC REED stepped-wedge cluster randomised trial
Source: BMJ Open. 2025 Aug 16;15(8):e104715. doi: 10.1136/bmjopen-2025-104715 (PMC12359426; doi:10.1136/bmjopen-2025-104715)
Supplement: online supplemental file 3 [file bmjopen-15-8-s003.pdf]

Document d'information à l'attention du patient
POUR LA POURSUITE DE L'ETUDE APRES SITUATION D'URGENCE
Patient en capacité de recevoir l'information aux urgences

IMPACT D'UNE STRATEGIE DE PRISE EN CHARGE DE LA DYSPNEE AIGUE CHEZ LE SUJET AGE BASEE SUR L'UTILISATION DE L'ECHOGRAPHIE CARDIOPULMONAIRE
LUC-REED_ RC31/23/0386

Promoteur de la recherche : CHU de TOULOUSE
Investigateur Coordonnateur : Dr Frédéric BALEN

PARTIE 1 : INFORMATIONS SUR LA RECHERCHE

Madame, Monsieur,

Lors de votre admission dans le service des urgences, en raison de la gravité de votre état de santé et de l'urgence médicale nous avons été dans l'impossibilité de vous demander votre approbation préalable pour la participation à cette étude. Nous vous avons donc inclus en demandant l'autorisation à votre proche ou personne de confiance ou en son absence, en utilisant la procédure d'urgence, le (date) |__|__| |__|__| |__|__|.

Conformément à l'article L1122-1-3 du Code de la Santé Publique, nous sollicitons maintenant votre accord pour la poursuite de votre participation à une recherche impliquant la personne humaine ayant pour objectif d'évaluer l'impact de l'échographie cardiopulmonaire dans le diagnostic de la dyspnée (difficulté à respirer) dont vous êtes atteint.

Vous êtes libre de participer ou non. Vous pouvez prendre le temps nécessaire pour lire les informations ci-dessous, discuter avec vos proches et votre médecin traitant et poser toutes vos questions au médecin de la recherche, appelé investigateur. Après avoir obtenu les réponses satisfaisantes à vos questions et disposé d'un délai de réflexion minimum d'une heure, vous pourrez alors décider si vous acceptez de continuer de participer à la recherche ou non.
Si vous ne souhaitez pas participer à cette étude, les données jusqu'alors enregistrées pour l'étude ne seront pas utilisées et seront détruites et vous continuerez à bénéficier de la meilleure prise en charge médicale possible, conformément aux connaissances actuelles.

<u>Pourquoi cette recherche est-elle mise en place ?</u>

La difficulté à respirer, aussi appelée dyspnée, est un motif fréquent et grave de recours aux structures d'urgences. Ce symptôme pose une difficulté de diagnostic précoce car son origine peut être variée (cardiologique, pulmonaire, infectieuse, …). Cette problématique retarde la mise en place d'une prise en charge thérapeutique adaptée. Il a néanmoins été démontré que lorsque le patient recevait une thérapeutique adaptée au plus tôt de sa prise en charge, cela avait un retentissement non négligeable sur son rétablissement.

Les examens complémentaires, en particulier biologiques, améliorent la prise en charge et le devenir des malades dyspnéiques aux urgences. Cependant ils ne sont pas disponibles immédiatement, incitant le médecin, au vu de la gravité du patient, à initier des traitements sans attendre leur(s) résultat(s). Cela peut donc engendrer la prescription de thérapeutiques inappropriés.
L'échographie pulmonaire réalisée par le médecin urgentiste, disponible immédiatement au lit du patient, pourrait permettre de réduire le temps pour poser un diagnostic et donc d'initier plus rapidement la meilleure thérapeutique.

<u>En quoi la recherche consiste-t-elle ?</u>

Pour participer à cette étude, vous devez être admis dans un service d'urgences avec les critères principaux suivants :

- Avoir plus de 65 ans
- Présenter une difficulté pour respirer (dyspnée)

L'objectif de cette recherche est d'évaluer l'impact de l'utilisation de l'échographie cardiopulmonaire sur la prise en charge thérapeutique initiale des sujets âgés consultant pour difficultés pour respirer en structures d'urgences.
Une analyse médico-économique sera également effectuée en parallèle afin d'évaluer l'impact budgétaire (coûts médicaux) de l'introduction de l'échographie cardiopulmonaire sur le séjour du patient dans la structure de soin. Ces données seront recueillies auprès de Caisse Nationale d'Assurance Maladie.

La durée de participation de chaque patient est de 30 jours, comprenant la durée de passage aux urgences, d'hospitalisation selon l'orientation décidée par le médecin et d'un rappel à 30 jours par un personnel de recherche.
Cette étude est réalisée dans 7 services d'urgences en France. Un total de 504 patients devra être recruté.
Cette étude a reçu un financement provenant du programme hospitalier de recherche clinique inter-régional de 2023 (PHRCi).

<u>Quelles est la stratégie étudiée ?</u>

La recherche se divise en deux périodes pour chaque service participant avec une période « contrôle » et une période « intervention ».
La prise en charge des patients inclus durant la première période est réalisée conformément aux recommandations et aux habitudes du service.
Au cours de la seconde période, les patients du service seront pris en charge selon les recommandations de la recherche, c'est-à-dire, avec la réalisation précoce d'une échographie cardio-pulmonaire au lit du malade en plus de la prise en charge classique dans un service d'urgence.

L'échographie pleuro-pulmonaire est une technique d'imagerie rapide (environ 5 minutes) et non invasive permettant de visualiser le poumon et d'identifier si ce dernier est normal ou présente une pathologie. Cet examen peut être réalisé au lit du malade à l'aide d'un appareil d'échographie portable par le médecin urgentiste.

Au démarrage de l'étude, tous les centres appliqueront la prise en charge habituelle. Puis, l'un après l'autre, les centres passeront dans la période « intervention » avec application des recommandations de la recherche. A la fin, tous les centres seront entrés dans la seconde période.

<u>Comment la recherche se déroule-t-elle ?</u>

Lors de votre consultation au sein du service des urgences pour difficultés à respirer, le médecin urgentiste a procédé à votre inclusion dans l'étude auprès de votre proche ou personne de confiance ou via la procédure d'urgence si personne n'était présent lors de votre admission après vérification des critères d'inclusions et de non inclusion. Il a ensuite procédé au recueil des données démographiques et cliniques.

Si vous avez été inclus lors de la première période, votre prise en charge est celle qui est mise en place habituellement dans ce type de pathologie. Un examen clinique est réalisé ainsi qu'une prise de sang et un examen radiographique, pouvant se traduire soit par une radiographie pulmonaire soit par un scanner pulmonaire selon la décision du médecin. Si vous avez été inclus lors de la seconde période dite « intervention », le médecin urgentiste formé à la technique d'échographie réalise une échographie cardio-pulmonaire précoce au lit du malade en plus des examens routiniers (examen clinique, prise de sang, examen radiographique) afin d'optimiser son choix de prise en charge thérapeutique précoce.

A la sortie du service des urgences, l'investigateur recueille les informations concernant les thérapeutiques entreprises lors du passage dans le service, les résultats des examens complémentaires, l'orientation du patient après le service d'urgence, ainsi que la durée de prise en charge.

Un mois après votre passage aux urgences, vous serez contacté par une personne du service (attaché de recherche clinique ou médecin investigateur) pour un entretien téléphonique d'environ 30 minutes afin de relever les hospitalisations qui ont eu lieu depuis votre sortie du service des urgences et de répondre à des questionnaires permettant l'analyse médico-économique et de qualité de vie. Le questionnaire médico-économique comporte 3 items reprenant votre parcours scolaire et professionnel. Celui de qualité de vie, le EQ-5D-5L, est une échelle de qualité de vie européenne avec 5 questions et une évaluation de l'état de santé actuel.

A l'issue de l'étude, un comité d'expert analysera tous les dossiers anonymisés des patients inclus dans l'étude sans connaître la période pendant laquelle le patient a été pris en charge afin de déterminer l'impact de l'utilisation de l'échographie cardiopulmonaire sur la prise en charge thérapeutique initiale.

Pour les besoins de l'analyse économique, des données relatives aux éventuelles prestations sanitaires auxquelles vous aurez eu recours durant la durée de l'étude (hospitalisations, consultations en ambulatoires, actes médicaux et paramédicaux, traitements, transports sanitaires éventuels) seront recueillies directement auprès de la Caisse Nationale d'Assurance Maladie. Pour cela, votre numéro de sécurité sociale (i.e. Numéro d'Inscription au Répertoire (NIR)), votre date de naissance complète et votre genre seront recueillis sur un fichier contenant un algorithme de cryptage instantané

conformément aux conditions prévues par la Commission Nationale de l'Informatique et des Libertés (CNIL).

Quelles sont les éventuelles alternatives médicales ?

Vous êtes libre de refuser de participer à cette étude ainsi que de mettre un terme à votre participation à n'importe quel moment, sans devoir vous justifier et sans que cela n'entraîne de conséquence sur la qualité des soins qui vous seront prodigués. Votre prise en charge sera conforme aux recommandations.

Quels sont les bénéfices attendus et risques liés à la recherche ?

Cette étude pourrait prouver le bénéfice diagnostic de l'utilisation précoce de l'échographie chez les patients souffrant de dyspnées avec une prise en charge plus rapide et plus spécifique.

Il n'existe pas de risques liés à l'utilisation de la machine d'échographie. L'innocuité de l'examen échographique et la rapidité d'exécution (inférieur à 6 minutes) en fait un examen complémentaire de choix au lit du malade.

Que se passera-t-il en cas d'arrêt prématuré de la recherche et après la recherche ?

En fin de recherche la prise en charge continuera conformément aux recommandations. L'exclusion de l'étude ou l'arrêt prématuré de l'étude ne modifie pas la prise en charge globale de la pathologie.

Durée de conservation des données

Seules les informations strictement nécessaires au traitement et à la finalité de la recherche seront recueillies et ces données seront conservées au maximum 2 ans après la dernière publication ou jusqu'à la signature du rapport final puis archivées pendant 15 ans.

Dispositions législatives et réglementaires

Conformément à l'article L. 1121-4 du Code de la santé publique, cette recherche a obtenu un avis favorable du Comité de Protection des PersonnesCPP EST VI en date du 25/07/2024] et a fait l'objet d'une information à l'Agence Nationale de Sécurité du Médicament et des produits de santé (ANSM).
Le traitement de vos données personnelles dans le cadre de la recherche bénéficie d'une autorisation de la CNIL.
Pour couvrir sa responsabilité et celle de toute personne intervenant dans la réalisation de la recherche, en vertu de l'article L. 1121-10 du CSP, le CHU de Toulouse de cette recherche a souscrit une assurance de responsabilité civile auprès de la société Lloyd's Insurance Company SA (n° contrat d'assurance : HSLCET24004).
Le cas échéant, une convention a été établie entre l'établissement de santé et le promoteur, lequel prend en charge les frais supplémentaires et contreparties financières engendrées par la recherche.

PARTIE 2 : INFORMATIONS SUR LES DROITS DU PARTICIPANT ET SUR LA GESTION DES DONNEES RECUEILLIES

Que signifie le principe d'un consentement libre et éclairé à la participation à une recherche impliquant la personne humaine ?

La participation à une recherche impliquant la personne humaine est libre et volontaire : vous êtes libre d'accepter ou de refuser de participer à cette étude et vous pouvez interrompre à tout moment votre participation sans avoir à donner de raison et sans encourir aucune responsabilité ni préjudice de ce fait. Il vous suffit de le signaler à l'investigateur.
Votre décision de participer ou de ne pas participer n'aura aucune conséquence sur votre prise en charge médicale et la qualité de vos soins ou sur votre relation avec l'investigateur.
Pour continuer de participer à cette recherche, vous devez donner préalablement votre consentement libre et éclairé. « Eclairé » signifie que vous aurez bénéficié d'une information claire et compréhensible sur les enjeux et le déroulement de la recherche et sur vos droits en tant que participant.
Vous serez informé(e) par l'investigateur qui vous suit de toute nouvelle information concernant la recherche qui pourrait modifier votre décision.

Vous avez le droit d'obtenir communication, au cours ou à l'issue de la recherche, des informations concernant votre santé, détenues par l'investigateur ou, le cas échéant, le médecin ou la personne qualifiée qui le représente.

Si vous le souhaitez, vous pourrez être informé(e) des résultats globaux de cette recherche conformément aux dispositions de l'article L. 1122-1 du code de la santé publique, une fois que celle-ci sera achevée, en contactant l'investigateur qui vous a proposé l'étude.

Comment vos données personnelles seront-elles traitées dans le cadre de la recherche?

Si vous acceptez de participer à la recherche, vos données personnelles, y compris vos données de santé, feront l'objet d'un traitement par le promoteur, en qualité de responsable du traitement de ces données.
Les données suivantes seront recueillies :
- Données démographiques
- Antécédents médicaux
- Données cliniques, biologiques et d'imagerie recueillies lors de votre passage aux urgences
- Suivi à J30 (hospitalisation, décès, qualité de vie)
- Numéro de sécurité sociale

Quelle est la base juridique et la finalité du traitement de vos données personnelles ?

Le traitement de vos données personnelles est nécessaire à la réalisation de la recherche et est fondé sur la mission d'intérêt public dont est investi le promoteur.
Ce traitement est autorisé car il est nécessaire à des fins de recherche scientifique. Le responsable de traitement doit mettre en œuvre des mesures appropriées permettant de garantir vos droits et libertés, notamment le seul recueil de données strictement nécessaires à la recherche.

Comment la confidentialité de vos données sera-t-elle assurée ?

Vos données personnelles seront traitées de manière confidentielle, conformément à la loi du 6 janvier 1978 modifiée dite « Loi Informatique et Libertés », et conformément au Règlement Général sur la Protection des Données (RGPD).

Vos données seront codées, c'est-à-dire que vous serez identifié(e) par un numéro de code pour les besoins de la recherche, sans mention de vos noms et prénoms. Seul l'investigateur conservera la liste de correspondance entre le code et votre nom.

Qui aura accès à vos données dans le cadre de la recherche ?

Les informations concernant votre identité (nom, prénom) ne seront connues que par l'équipe médicale vous prenant en charge ainsi que par les personnes réalisant le contrôle de la qualité de la recherche mandatées par le promoteur, par les autorités sanitaires ou de contrôle, par le délégué à la protection des données du promoteur si vous le contactez à l'adresse dpo@chu-toulouse.fr et, en cas de litige, par le personnel habilité de l'organisme d'assurance du promoteur.
Ces personnes sont soumises au secret professionnel.

Vos données codées seront accessibles aux personnes suivantes :

- Le promoteur et les personnes agissant pour son compte,
- Les experts indépendants chargés de ré-analyser les données pour vérifier les résultats de la recherche, en vue de leur publication, dans des conditions strictes de sécurité.

Ces personnes, soumises au secret professionnel, auront accès à vos données codées dans le cadre de leur fonction et en conformité avec la réglementation.

Quels sont vos droits relatifs à vos données personnelles ?

Vous avez le droit d'accéder à vos données, par l'intermédiaire de l'investigateur, et demander à ce qu'elles soient rectifiées ou complétées.
Vous pouvez également demander la limitation du traitement de vos données (c'est-à-dire demander au promoteur de geler temporairement l'utilisation de vos données).
Même si vous acceptez de participer à la recherche, vous pourrez à tout moment vous opposer au traitement de vos données aux fins de réalisation de la recherche. Dans ce cas, aucune information supplémentaire vous concernant ne sera collectée.
Vous pouvez également exercer votre droit à l'effacement sur les données déjà recueillies mais celles-ci pourront ne pas être effacées si cela rendait impossible ou compromettait gravement la réalisation des objectifs de la recherche.

De plus, certaines données visant à assurant la qualité et la sécurité de la recherche (par exemple : les effets indésirables des produits testés) doivent obligatoirement être collectées par le promoteur. Vous ne pourrez pas exercer votre droit d'opposition ou d'effacement concernant ces données.

Vous pouvez également accéder directement ou par l'intermédiaire d'un médecin de votre choix à l'ensemble de vos données médicales en application des dispositions de l'article L. 1111-7 du Code de la Santé Publique.

Comment exercer vos droits ?

Vous pouvez exercer vos droits à tout moment et sans avoir à vous justifier.

Le promoteur n'ayant pas accès à votre identité, il est recommandé de vous adresser, dans un premier temps, à l'investigateur, aux coordonnées disponibles dans la présente note.
Vous pouvez en outre, si vous le souhaitez, exercer vos droits auprès du délégué à la protection des données du promoteur par l'adresse dpo@chu-toulouse.fr qui gèrera cette demande en coordination avec le médecin et les professionnels impliqués dans l'étude. Dans ce cas, votre identité (prénom, nom) sera rendue accessible au délégué à la protection des données du promoteur.

Dans l'hypothèse où vous ne parvenez pas à exercer vos droits, vous disposez également du droit de déposer une réclamation concernant le traitement de vos données personnelles auprès de la Commission nationale de l'informatique et des libertés (CNIL), qui est l'autorité de contrôle compétente en France en matière de protection des données.

Vos données codées pourront-elles être réutilisées ?

Vous pouvez accepter ou refuser le principe de l'utilisation de vos données codées lors de recherches ultérieures, conduites exclusivement à des fins scientifiques dans le domaine de la dyspnée et de l'échographie médicale.

Si vous en acceptez le principe, vous serez recontacté(e) pour être informé(e) des caractéristiques du / des nouveaux traitement(s) conformément à l'article 14 du RGPD, et si vous ne vous y opposez pas, vos données codées pourront être réutilisées et transmises pour ces autres projets de recherche dans le domaine de la santé.
Cette/ces recherche(s) ultérieure(s) devra(ont) soit être conforme(s) à un référentiel établi par la CNIL si elle(s) entre(nt) dans le cadre d'une procédure simplifiée du fait de ses/leurs caractéristiques, soit faire l'objet d'une autorisation de la CNIL.

Grâce à cette information, vous pourrez choisir d'exercer vos droits d'accès, de rectification, de limitation, d'opposition ou d'effacement de vos données. L'affichage des projets sur la page du site internet vaut information pour cette réutilisation de données et dès lors, il n'est pas prévu de vous adresser une lettre individuelle d'information complémentaire pour chaque projet de recherche.
Les modalités d'opposition pour chaque projet de recherche seront indiquées sur ce site Internet.

Toute l'équipe vous remercie et se tient à votre disposition pour répondre à vos questions.

Vos contacts

Promoteur de la recherche et responsable du traitement	CHU de Toulouse, Hôtel-Dieu, 2 rue Viguerie, 31052 Toulouse cedex 9<br>www.chu-toulouse.fr
Investigateur coordonnateur de la recherche	Dr BALEN Frédéric<br>Pole Médecine d'Urgence ; CHU Toulouse ;<br>1 place Baylac ; 31058 Toulouse<br>balen.f@chu-toulouse.fr
Investigateur principal du lieu de recherche	Coordonnées postales, téléphone et mail (à compléter par le centre)
Délégué à la protection des données	Délégué à la protection des données du CHU de Toulouse _Hôpital Rangueil - TSA 50032 – 31059 Toulouse Cedex<br>DPO@chu-toulouse.fr
CNIL- Commission nationale de l'Informatique et des libertés	CNIL - 3 Place de Fontenoy - TSA 80715 - 75334 PARIS CEDEX 07<br><br>www.cnil.fr<br>**Déposer une plainte en ligne :**<br>https://www.cnil.fr/fr/webform/adresser-une-plainte.<br>.

PARTIE 3 : GLOSSAIRE

**Recherche Impliquant la personne humaine**	Les recherches organisées et pratiquées sur l'être humain en vue du développement des connaissances biologiques ou médicales sont désignées par le terme « recherche impliquant la personne humaine » (article L. 1121-1 du Code de la santé publique)
**Promoteur**	Personne physique ou morale responsable de la recherche, qui en assure la gestion et qui vérifie que son financement est prévu.
**Investigateur**	Personne physique chargée de surveiller et de diriger la recherche sur un lieu de recherche.
**RGPD**	Règlement Général sur la Protection des Données.<br>Règlement (UE) 2016/679 du Parlement européen et du Conseil du 27 avril 2016 relatif à la protection des personnes physiques à l'égard du traitement des données à caractère personnel et à la libre circulation de ces données
**Étude randomisée**	Étude au sein de laquelle l'attribution dans un groupe se fait de façon aléatoire par tirage au sort.
**Placebo**	Substance sans principe actif.
**Résultats globaux**	Résultats de la recherche résultant de l'analyse de toutes les données de celle-ci.
**Données personnelles**	Donnée se rapportant à une personne physique identifiée ou identifiable. Les données de santé sont des données à caractère personnel particulières car sensibles.
**Données codées Ou pseudonymisées**	Le codage ou la pseudonymisation consiste à remplacer les données directement identifiantes (nom, prénom, etc.) d'un jeu de données par des données indirectement identifiantes (alias, numéro, etc.).
**Méthodologie de référence (MR)**	Procédure simplifiée encadrant l'accès aux données de santé pour les promoteurs de recherche
**Traitement des données**	Un traitement de données personnelles est une opération, ou ensemble d'opérations, portant sur des données personnelles, quel que soit le procédé utilisé (collecte, enregistrement, organisation, conservation, adaptation, modification, extraction, consultation, utilisation, communication par transmission ou diffusion ou toute autre forme de mise à disposition, rapprochement).

Formulaire de consentement poursuite pour le patient
Patient en capacité de recevoir l'information aux urgences

Impact d'une stratégie de prise en charge de la détresse respiratoire aiguë chez le sujet âgé basée sur l'utilisation de l'échographie cardiopulmonaire
LUC-REED

Promoteur : CHU de Toulouse
Investigateur Coordonnateur : Dr Frédéric Balen

Je soussigné(e) [*Nom, Prénom*] consens librement à participer à cette recherche telle que décrite dans la lettre d'information et je confirme les points suivants :

- J'ai eu le temps de lire ces informations, de réfléchir à l'étude et j'ai obtenu des réponses appropriées à mes questions.
- J'ai bien été informé de la nature des objectifs de la recherche, des risques potentiels et des contraintes liées à cette recherche.
- Je certifie être affilié(e) à un régime de sécurité sociale ou bénéficiaire d'un tel régime, sauf dérogation exceptionnelle.
- J'ai le droit de refuser de participer à la recherche ou de retirer mon consentement à tout moment sans conséquence sur ma prise en charge médicale et sans encourir aucune responsabilité ni préjudice de ce fait.
- J'ai bien compris la possibilité qui m'est réservée d'interrompre ma participation à cette recherche à tout moment sans avoir à justifier ma décision et j'informerai l'investigateur qui me suit dans la recherche. Cela ne remettra pas en cause la qualité des soins ultérieurs.
- J'ai bien compris que l'investigateur peut interrompre à tout moment ma participation à la recherche s'il le juge nécessaire.
- J'ai bien noté que je dispose d'un droit d'accès, de rectification, de limitation et, le cas échéant, d'opposition et d'effacement, concernant le traitement de mes données personnelles. Ces droits s'exercent en premier lieu auprès de l'investigateur qui me suit dans le cadre de cette recherche et qui connaît mon identité.
- J'ai bien pris connaissance que cette recherche a reçu l'avis favorable du Comité de Protection des Personnes CPP EST IV en date du 25/07/2024 et a fait l'objet d'une information auprès de l'Agence Nationale de Sécurité du Médicament. Le promoteur de la recherche a souscrit une assurance de responsabilité civile en cas de préjudice auprès de la société Lloyd's Insurance Company SA (n° contrat d'assurance : HSLCET24004).
- Mon consentement ne décharge en rien l'investigateur et le promoteur de la recherche de leurs responsabilités à mon égard. Je conserve tous mes droits garantis par la loi.
- Les résultats globaux de la recherche me seront communiqués à la fin de la recherche, si j'en fais la demande auprès de l'investigateur.
- En cas d'examen susceptible de déceler des anomalies, je consens à être tenu informé(e) des informations relatives à mon état de santé et des éventuelles anomalies qui pourraient être décelées à l'occasion de la recherche.

- Après le commencement de la recherche, je pourrais à tout moment demander des informations complémentaires au médecin qui me suit dans la recherche.
- Deux exemplaires originaux de ce formulaire de consentement ont été établis : un m'a été remis, le second gardé par l'investigateur. Ils seront conservés dans le dossier de l'étude au minimum 15 ans après la fin de la recherche.
- J'ai été informé(e) sur la façon dont mes données personnelles pourront être collectées, utilisées et partagées comme décrit dans ce document.

En cas de nécessité pour la continuité de mes soins et mon suivi, j'accepte que mon médecin traitant soit informé de ma participation à cette recherche	Oui ☐	Non ☐
J'accepte que mes données personnelles codées soient utilisées pour d'autres recherches liées à la santé ou à la médecine, exclusivement à des fins scientifiques sachant que je peux à tout moment retirer mon accord.	Oui ☐	Non ☐

Signature du/de la participant(e)	Date de signature
Prénom et Nom du/de la participant(e) en majuscules	Date de naissance pour éviter les homonymies
Signature de l'investigateur ou du médecin / professionnel de santé qui le représente ayant informé le/la participant(e)	Date de signature
Prénom et nom de l'investigateur ou du médecin / professionnel de santé qui le représente ayant informé le/la participant(e)	
